# Human Movement Representation on Multivariate Time Series for Recognition of Professional Gestures and Forecasting Their Trajectories

**DOI:** 10.3389/frobt.2020.00080

**Published:** 2020-08-13

**Authors:** Sotiris Manitsaris, Gavriela Senteri, Dimitrios Makrygiannis, Alina Glushkova

**Affiliations:** Centre for Robotics, MINES ParisTech, PSL Université, Paris, France

**Keywords:** state-space representation, differential equations, movement modeling, hidden Markov models, gesture recognition, forecasting, motion trajectory

## Abstract

Human-centered artificial intelligence is increasingly deployed in professional workplaces in Industry 4.0 to address various challenges related to the collaboration between the operators and the machines, the augmentation of their capabilities, or the improvement of the quality of their work and life in general. Intelligent systems and autonomous machines need to continuously recognize and follow the professional actions and gestures of the operators in order to collaborate with them and anticipate their trajectories for avoiding potential collisions and accidents. Nevertheless, the recognition of patterns of professional gestures is a very challenging task for both research and the industry. There are various types of human movements that the intelligent systems need to perceive, for example, gestural commands to machines and professional actions with or without the use of tools. Moreover, the *inter*class and *intra*class spatiotemporal variances together with the very limited access to annotated human motion data constitute a major research challenge. In this paper, we introduce the Gesture Operational Model, which describes how gestures are performed based on assumptions that focus on the dynamic association of body entities, their synergies, and their serial and non-serial mediations, as well as their transitioning over time from one state to another. Then, the assumptions of the Gesture Operational Model are translated into a simultaneous equation system for each body entity through State-Space modeling. The coefficients of the equation are computed using the Maximum Likelihood Estimation method. The simulation of the model generates a confidence-bounding box for every entity that describes the tolerance of its spatial variance over time. The contribution of our approach is demonstrated for both recognizing gestures and forecasting human motion trajectories. In recognition, it is combined with continuous Hidden Markov Models to boost the recognition accuracy when the likelihoods are not confident. In forecasting, a motion trajectory can be estimated by taking as minimum input two observations only. The performance of the algorithm has been evaluated using four industrial datasets that contain gestures and actions from a TV assembly line, the glassblowing industry, the gestural commands to Automated Guided Vehicles as well as the Human–Robot Collaboration in the automotive assembly lines. The hybrid approach State-Space and HMMs outperforms standard continuous HMMs and a 3DCNN-based end-to-end deep architecture.

## Introduction

Human motion analysis and recognition are widely researched from various scientific domains including Human–Computer Interaction, Collaborative Robotics, and Autonomous Vehicles. Both the industry and science face significant challenges in capturing the human motion, developing models, and algorithms for efficiently recognizing it, as well as for improving the perception of the machines when collaborating with humans.

Nevertheless, in factories, “we always start with manual work,” as explained by Mitsuri Kawai, Head of Manufacturing and Executive Vice-President of Toyota (Borl, 2018). Therefore, experts from both collaborative robotics and applied ergonomics are always involved when a new collaborative cell is being designed. Nowadays, despite the significant progress in training robots by demonstration, automatizing the human tasks in mixed workspaces still remains the goal. However, those workspaces are not necessarily collaborative. For example, in a smart workspace, a machine that perceives and anticipates gestures and actions of the operator would be able to adapt its own actions depending on those of the operator, thus giving him/her the possibility to obtain ergonomically “green postures.” Furthermore, automated guided vehicles (AGVs) should also be able to detect the intentions of the operator with the aim of collaborating with them, avoiding accidents and understanding gestural commands. Finally, in Industry 4.0, an important number of Creative and Cultural Industries, for example, in luxury goods manufacturing, still base their know-how on manual dexterity, no matter whether the operator is in collaboration with a machine or not. Therefore, human movement representation and gesture recognition constitute a mean for identifying the industrial know-how and transmitting it to the next generation of the operators.

From a scientific point of view, major research challenges are faced by scientists, especially when dealing with professional environments in an industrial context. Initially, there is an extremely limited access to motion data from real-life configurations. This is mainly due to acceptability issues from the operators or to limitations imposed by laws and regulations that protect the access to/use of personal data, for example, the “General Data Protection Regulation” in the European Union. Therefore, most of existing datasets include only gestures from the everyday life. Furthermore, when creating custom datasets with professional motion data, many practical, and environmental issues might occur, for example, variation in luminosity, various workspace with different geometries, camera in motion to record a person moving in space, and low availability of real experts. Additionally, the community of actions and gesture recognition deals with challenges that are related to intraclass and *inter*class variations (Fu, 2016). Frequent are the cases where a professional task involves gestures that have very similar spatiotemporal characteristics (*low interclass variation*) together with very important differences in the way different humans perform the same gesture (*high intraclass variation*). Finally, when applied to accident prevention, a small delay in predicting the action might be crucial.

The work presented in this paper contributes to the aforementioned challenges, through the proposition of a Gesture Operational Model (GOM) that describes how the body parts cooperate, to perform a situated professional gesture. The model is built upon several assumptions that determine the dynamic relationship between the body entities within the execution of the human movement. The model is based on the State-Space (SS) representation, as a simultaneous equation system for all the body entities is generated, composed of a set of first-order differential equations. The coefficients of the equation system are estimated using the Maximum Likelihood Estimation (MLE) method, and its dynamic simulation generates a dynamic tolerance of the spatial variance of the movement over time. The scientific evidence of the GOM is evaluated through its ability to improve the recognition accuracy of gestural time series that are modeled using continuous Hidden Markov Models (HMMs). Moreover, the system is dynamically simulated through the solution of its equations. Its forecasting ability is evaluated by comparing the similarity between the real and simulated motion data using two real observations for initializing the models as well as by measuring the Theil inequality coefficient and its decompositions.

The performance of the algorithms that implement the GOM, the recognition of gestures, and the forecasting of the motion trajectories are evaluated by recording four industrial real-life datasets from a European house-holding manufacturer, a glassblowing workshop, an AGV manufacturer, and a scenario in automotive industry. More precisely, the first dataset contains motion data with gestures and actions from a TV assembly line, the second from the creation of glass water carafes, the third gestural commands to mobile robots, and the fourth from a scenario of Human–Robot Collaboration in the automotive industry. The motion data used in our experiments are 2D positions that are exported from computer vision and the application of a deep-learning-based pose estimation using the OpenPose framework (Cao et al., 2019).

*State of the Art* presents a state of the art on human motion modeling, representation, and recognition. In *Methodology*, the whole methodology analysis, modeling, and recognition are presented, whereas in *Evaluation*, the different approaches in the evaluation of the ability of the models to simulate a gesture and forecast its trajectories are analyzed. In the same section, the accuracy of the proposed method is also presented. Finally, in *Discussion* and *Conclusion and Future Work*, a discussion and the future work and perspectives of the proposed methodology are described.

## State of the Art

In professional environments, a movement can be defined as the displacement of the human body in space, whereas gesture is a form of non-verbal communication for interacting with a machine or manipulating a tool or object. In industry, professional gestures define the routine of workers. The non-physical interaction of the workers with collaborative machines is relying on an external perception layer that gets input from the ambient environment using, among others, human motion sensors, to understand their movement and adapt their behavior according to the humans. Whether machine or deep learning can be used to create an AI-based perception layer for collaborative machines. Machine learning has demonstrated an important number of applications in action and gesture recognition by using whether a probabilistic modeling of the phenomenon, and optionally a representation of it, or a template matching that makes use of a temporal rescaling of the input signal according to the reference. Finally, architectures of deep neural networks have recently seen a considerable number of applications with a high accuracy and precision.

### Movement Modeling and Representation

Each body articulation is strongly affected by the movement of other articulations. Observing a person running brings evidence on the existing interdependencies between different parts of human body that need to move cooperatively for a movement to be achieved. Duprey et al. (2017) attempted to study those relationships by exploring the upper body anatomy models available and describe their applicability using multi-body kinematic optimization, mostly for clinical, and ergonomic uses. Biomechanics has also actively contributed to the study of human movement modeling by using Newtonian methods and approaches, especially in sports and physical rehabilitation (Zatsiorsky, 2000). The representation of the human movement with physical or statistical models provides with a simplified mathematical formalization of the phenomenon and approximates reality, e.g. through simulation and forecasting. State-Space (SS) is a statistical modeling that allows to stochastically represent a human movement through a reasoning over time that makes use of internal states. An SS representation is a mathematical model of a physical system as a set of input, output, and state variables related by first-order differential equations. Kalman filtering is a method that estimates and determines values for the parameters of a model. To represent human movement, Zalmai et al. (2015) used linear SS models and provided an algorithm based on local likelihood for detecting and inferring gesture causing magnetic field variations. Lech and Kostek (2012) used Kalman filtering to achieve hand tracking and presented a system based on camera and multimedia projector enabling a user to control computer applications by dynamic hand gestures. Finally, Dimitropoulos et al. (2016) presented a methodology for the modeling and classification of multidimensional time series by exploiting the correlation between the different channels of data and the geometric properties of the space in which the parameters of the descriptor lie by using a linear dynamic system (LDS). Here, multidimensional evolving data were considered as a cloud of points (instead of a single point) on the Grassmann manifold, and codebook is created to represent each multidimensional signal as a histogram of Grassmannian points, which is not always the case for professional gestures.

### Machine Learning for Gesture Recognition

#### Template-Based Machine Learning

Template-based machine learning has been widely used for gesture recognition in the context of continuous real-time human–machine interaction. Dynamic Time Warping (DTW) is a template-based method that has been widely used for measuring the similarity between motion data. DTW makes it possible to find the optimal global alignment between two sequences. Bevilacqua et al. (2005), Bevilacqua et al. (2007), and Bevilacqua et al. (2009) successively developed a system based on DTW, the Gesture Follower, for both continuous gesture recognition and following, between the template or reference gesture, and the input or performed one. A single example allows the training of the system (Bobick and Wilson, 1997). During the performance, a continuous estimation of parameters is calculated in real time, providing information for the temporal position of the performed gesture. Time alignment occurs between the template and the performed gesture, as well as an estimation of the time progression within the template in real time. Instead, Psarrou et al. (2002) used the Conditional Density Propagation algorithm to perform gesture recognition and made sure that they will not get probabilities for only one model per time stamp. The experiments resulted to a relatively good accuracy for the time period conducted.

#### Model-Based Machine Learning

One of the most popular methods of model-based machine learning that has been used to model and recognize movement patterns are HMMs. Pedersoli et al. adopted this method (Pedersoli et al., 2014) to recognize in real-time static hand poses and dynamic hand gestures of American Sign Language. Sideridis et al. (2019) created a gesture recognition system for everyday gestures recorded with inertia measurement units, based on Fast Nearest Neighbors, and Support Vector Machine methods, whereas Yang and Sarkar (2006) chose to use an extension of HMMs. Vaitkevičius et al. (2019) used also HMMs with the same purpose, gesture recognition, for the creation of virtual reality installations, as well as Williamson and Murray-Smith (2002), who used a combination of HMMs with a dynamic programming recognition algorithm, along with the granular synthesis method for gesture recognition with audio feedback. In a more industrial context, Yang et al. (2007) used gesture spotting with HMMs to achieve efficient Human–Robot collaboration where real-time gesture recognition was performed with extended HMM methods like Hierarchical HMMs (Li et al., 2017). HMMs seem to be a solid approach, allowing to achieve satisfying results of gesture modeling and recognition and are suitable for real-time applications.

The aforementioned methodologies and research approaches permit the identification of what/which gesture is performed by giving a probability, but not how expressively the gesture has been performed. Caramiaux et al. (2015) extended the research, by implementing a sequential Monte Carlo technique to deal with expressivity. The recognition system, named Gesture Variation Follower, is being adapted to gesture expressive variations in real time. Specifically, in the learning phase, only one example per gesture is required. Then, in the testing (recognition) phase, time alignment is computed continuously, and expressive variations (such as speed and size) are estimated between the template and the performed gesture (Caramiaux, 2015; Caramiaux et al., 2015).

The model-based and template-based methods present an interesting complementarity and their combination in most of cases, give the possibility to achieve satisfying gesture recognition accuracy. However, when the probability given per class presents a high level of uncertainty, these methods need to be completed with an extra layer of control that will permit to take a final, more robust, decision about the probability of an observation to belong to each class. One of the goals of this work is to focus on the use of the SS method for human movement representation and modeling and to use this representation as the extra control layer to improve gesture recognition results.

### Deep Architectures for Action Recognition

Deep Learning (DL) is another approach with increasing scientific evidence in action and gesture classification. Mathe et al. (2018) presented results on hand gesture recognition with the use of a Convolutional Neural Network (CNN), which is trained on Discrete Fourier Transform images that resulted from raw sensor readings. In Oyedotun and Khashman (2017), the authors proposed an approach for the recognition of hand gestures from the American Sign Language using CNNs and auto-encoders. 3DCNNs are used in Molchanov et al. (2015) to detect hand movements of drivers, and in Camgoz et al. (2016) continuously recognize gesture classes from the Continuous Gesture Dataset (ConGD), which is the larger user-independent dataset. A two-stage approach is presented in Li et al. (2015), which combines feature learning from RGB-D using CNNs with Principal Component Analysis (PCA) for selecting the final features. Devineau et al. (2018) used a CNN model and tested its performance on classifying sequential humans' tasks using hand-skeletal data as input. Shahroudy et al. (2016), wanting to improve their action recognition results and decrease the dependency in factors like lightning, background, and color clothing, used a recurrent neural network to model the long-term correlation of the features for each body part. For the same reason, Yan et al. (2018) proposed a model of dynamic skeletons called spatial–temporal graph convolutional network (ST-GCN). This Neural Network (NN) learns automatically the spatial and temporal patterns from the given data, minimizing the computational cost, and increasing the generalization capability. In other cases, action recognition was achieved using either 3DCNNs (Tran et al., 2015) or two stream networks (Simonyan and Zisserman, 2014). CNNs are the NNs used in all cases above, as they are the main method used for image pattern recognition.

The particularity of DL methods is that they require a big amount of data in order to be trained. In some applications, having access to an important volume of data might not be possible for various reasons. One application with extremely limited amount of data is the recognition of situated professional actions and gestures performed in an industrial context, such as in manufacturing, assembling lines, and craftsmanship. Deep NNs are powerful methods for pattern recognition with great accuracy results, but they present some limitations for real-time applications, which are linked to the computational power that is required for both training and testing purposes. In this paper, given the fact that the available examples per gesture class are also limited, it is assumed and proved that stochastic model-based machine learning can give better results than DL.

The aim of this paper is to get advantage of existing knowledge in machine learning, and more precisely in the stochastic modeling for the recognition of gestures and the forecasting of their motion trajectories. To underline the advantages of the method proposed we also compare its performance with results obtained with a recent DL-based end-to-end architecture.

#### Our Previous Work

Manitsaris et al. (2015b) previously defined an operational model explaining how the body parts are related to each other, which was used for the extraction of confidence bounds over the time series of motion data. In Manitsaris et al. (2015b), as well as in Volioti et al. (2016), the operational model has been tested on Euler angles. In this work, the operational model is expanded to the full body and is tested with various datasets with position data from various real-life situations that have more classes and users than in previous work.

## Methodology

### Overview

**Figure 1:**
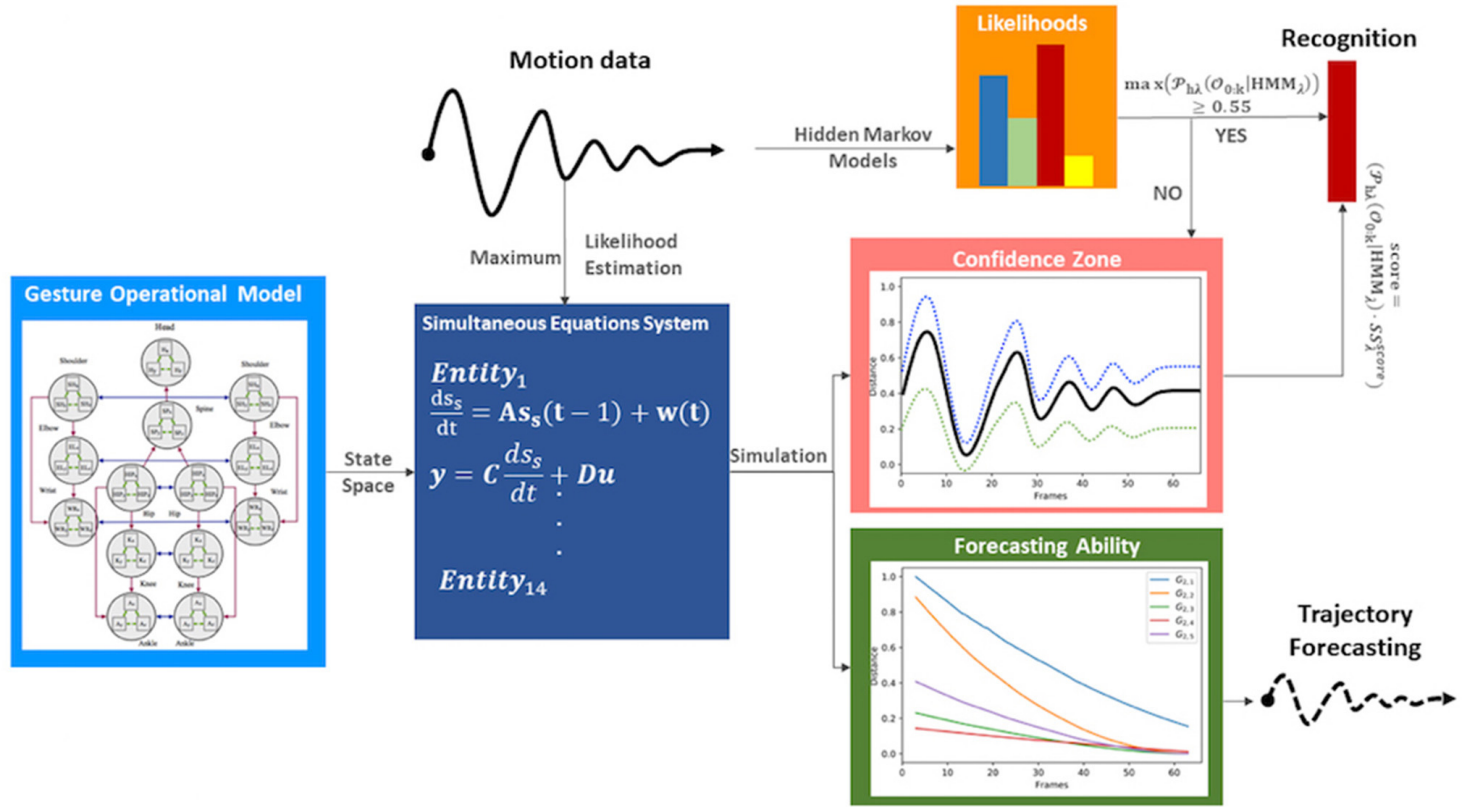
Methodology pipeline.

The motion capturing of the operators in their workplace is a major task. A number of professional gestural vocabularies are created to build the methodology and evaluate its scientific evidence. Although the proposed methodology (Figure 1) is compatible with various types of motion data, we opted for RGB sensors and, in most of cases, with 2D positions to avoid any interference between the operator and his/her tools or materials. Thus, RGB images are recorded for every gesture of the vocabulary, segmented into gesture classes, annotated, and then introduced to an external framework for estimating the poses and extracting the skeleton of the operators.

**Figure 2:**
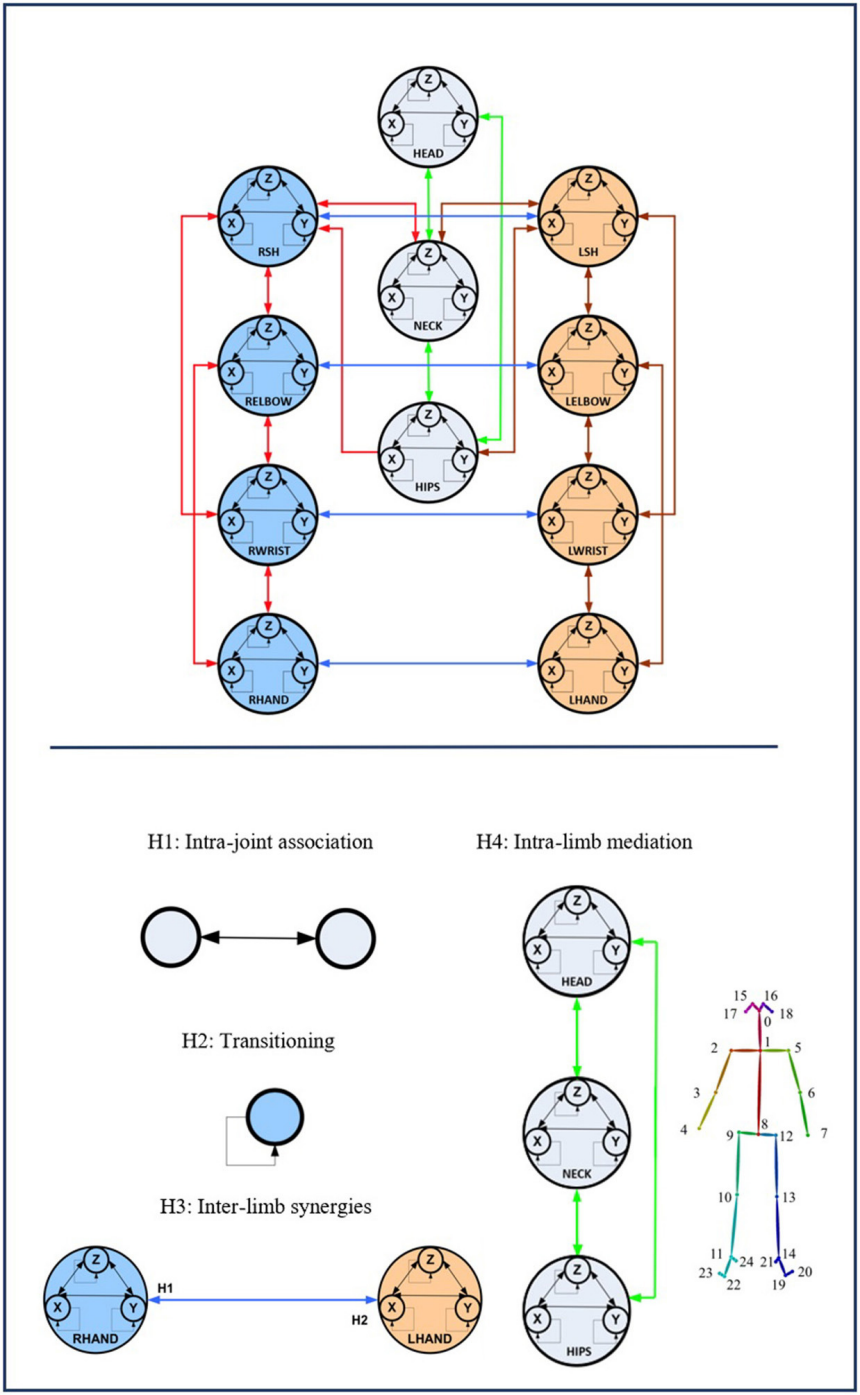
The full-body assumptions of the Gesture Operational Model (GOM) are depicted in the figure. Some relationships happen to be bidirectional, while others not. The relationships of the human body are governed by four different assumptions, intra-joint association, transitioning, inter-limb synergies, and intra-limb mediation. On the down-right of the image, the mapping on body is presented. The numbers in the GOM model, represent the corresponding body part of the joints representation from OpenPose framework. The idea of the GOM was based on a previous article of the authors (Manitsaris et al., 2015b)^1^.

As shown in Figure 2, the GOM is based on a number of assumptions that describe the way the different entities of the human body cooperate to efficiently perform the gesture. The assumptions of the model refer to various relationships between the entities, which are: the *intrajoint association*, the *interlimb synergies*, the *intralimb mediation*, and the *transitioning* over time. Following the theory of the SS modeling, the GOM is translated into a *simultaneous equation system* that is composed of two first-order differential equations for each component (e.g., dimension *X*, *Y* for 2D or *X* *Y*, *Z* for 3D) of each body entity.

During the training phase, the motion data of the training dataset are used to compute the coefficients of the equation system using the MLE method but also to execute a supervised learning of the continuous HMMs. Moreover, the motion data are used to solve the simultaneous equation system and simulate the whole gesture, thus generating values for the state variables. Once the solution of the system is completed for all the gestures of the vocabulary, the forecasting ability of every model is evaluated using the Theil coefficients as well as their performance in comparison with the motion data of other gestures.

During the testing phase, the HMMs output their likelihoods, which are multiplied by a confidence coefficient when their maximum likelihood is under a threshold. Finally, a motion trajectory can be dynamically or statically forecasted at any time by giving as input at least two time-stamp values from the real motion data.

### Industrial Datasets and Gesture Vocabularies

The performance of the algorithms is evaluated by recording four industrial real-life datasets from a house-holding manufacturer, a glassblowing workshop, an AGV manufacturer, and an automotive industry. For each dataset, a gesture vocabulary has been defined in order to segment the whole procedure into small human motion units (**Table 2**).

The first gesture vocabulary (*GV*_1_) includes four gestures where the operator takes the electronic card from one box and then takes a wire from another, connects them, and places them on the TV chassis. The gestures are performed in a predefined working space, in front of the conveyor and with the boxes placed on the left and right sides. However, the operator has a certain degree of variation in the way of executing the tasks, because the gestures are ample involving the whole body. Moreover, in order to avoid self-occlusions and scene occlusions, the camera is mounted on the top, which is not necessarily the optimal camera location for pose estimation algorithms, for example, OpenPose. Currently, in the factory, together with the operator who performs the actions of *GV*_1_, there is also a second operator who will be progressively replaced by a collaborative robot.

The second gestural vocabulary proposes gestural commands for controlling an AGV. This dataset *GV*_2_ contains five gestures involving mostly the arm and forearm. *G*_2,1_ initiates the communication with the AGV, by shaking the palm, whereas *G*_2,2_ and *G*_2,3_ turn left and right the AGV by raising the respective arms. *G*_2,4_ speeds up the AGV by raising three times the right hand, whereas *G*_2,5_ speeds down the AGV by rolling the right hand away from the hips with a distance of around 20/30 cm. All gestures of *GV*_2_ start and end with the *i*-pose.

The third gesture vocabulary *GV*_3_ contains four gestures performed by a glassblower when creating a water carafe. The craftsman executes the gestures in a very limited space that is defined by a specific metallic construction. The craftsman puts the pipe on the metallic structure and performs various manipulations of the glass by using tools, such as pliers. Three out of four gestures are performed while the craftsman is sitting. More precisely, he starts by shaping the neck of the carafe with the use of pliers (*G*_3,1_), then he tightens the neck to define the transition between the neck and the curved vessel (*G*_3,2_), he holds in his/her right hand a specific paper and shapes the curves of the blown part (*G*_3,3_), and finalizes the object and fixes the details by using a metallic stick (*G*_3,4_). In general, the right hand is manipulating the tools while the left is holding and controlling the pipe. In parallel with *G*_3,2_ and *G*_3,3_, an assistant is helping and blowing promptly the pipe to permit the creation of the blown curved part.

The last dataset (*GV*_4_) used in this paper is related to a real-life Human–Robot Collaboration scenario that has been recorded in the automotive assembly lines of PSA Peugeot Citroën (PSA Group). A dual-arm robot and the worker are facing each other in order to cooperate for assembling motor hoses. More precisely, for the assembling of the motor hoses, the robot gives to the worker one part from the right and one part from the left claw, and the worker takes two hose parts from the robot, joins them, screws them, and finally places the mounted motor hose in a box. In order for the robot to achieve the appropriate level of perception and move accordingly, it needs to make two specific actions “to take a piece in the right claw” and “to take a piece in the left claw.” Then, the worker can screw after the first gesture “to assemble” or can choose to screw later during the last assembly subtask. At the end of the assembly task, the worker puts the assembled piece in a box, which means that a cycle has just ended. Therefore, it is important for the robot to recognize the actions “to assemble” and “to screw” of the worker, so as to give at the correct moment the next motor piece with its arm. Twelve operators have been recorded in *GV*_4_.

The four datasets and vocabularies contain professional gestures performed in different industries and contexts. Important differences may be observed between them though. For example, *GV*_1_, *GV*_3_, and *GV*_4_ involve the manipulation of tools from the operator. Therefore, the distribution of variances alternates between high, for example, when moving for grabbing the tool, and low values, for example, when tools or objects are put on a specific position. In *GV*_1_, despite the fact that the gestures are performed in a predefined space, the operator has a certain degree of variation between different repetitions of the same gesture. Human factors such as the level of experience, fatigue, or even stress influence the way these gestures are performed without necessarily having a direct impact on the final result, which is to place the card on the TV. However, this is not the case of the *GV*_3_, where a high level of technicity and dexterity is required. In *GV*_3_, only low spatial and temporal variations can be accepted. The glass blower performs the gestures with a high repeatability from one repetition to another and successfully reproduces the object with exactly the same specifications, for example, size and diameter. The gestural commands of *GV*_2_ are simpler and ampler. A bigger freedom and variation are thus expected in the way they are performed. In *GV*_4_, the operator is performing actions with a high repeatability. Because the dual-arm robot Motoman SDA20 has been used, the operator, depending on whether he/she is left- or right-handed, has various possibilities for grabbing the parts from the robot and the tools.

In *GV*_1_, although all the gestures are performed by a single user, the different positions of the operator in space in each gesture make it an interesting dataset to work on. Also, this dataset appears to have a lot of noise, and it was an opportunity to examine the reaction of the pose estimation framework to noisy data. The second dataset (*GV*_2_) has multiple users, giving the opportunity to examine how gesture recognition works with a high variation among the performance of the same gestures. In the third gestural vocabulary (*GV*_3_), all gestures have been performed by an expert artist. They are fine movements where hands are cooperating in a synchronous way. Consequently, investigating body parts dependencies in this *GV* becomes extremely interesting. The fourth gestural vocabulary (*GV*_4_) has a robot involved in the industrial routine.

From an *intraclass* variance point of view, the Root-Mean-Square Error (RMSE) is used to evaluate the datasets. The RMSE allows the measurement of the difference between two times series, and it is defined as shown in Equation (1).

(1) $$\begin{array}{l} \text{RMSE}=\sqrt{\bar{{\left(o_{1}-o_{2}\right)}^{2}}} \end{array}$$

where *o*_1_ is one of the iterations of a specific gesture within a gestural vocabulary and *o*_2_ is another iteration of the same gesture, among which the variance is to be examined. A high variance between the iterations of each gesture of *GV*_2_ is noticed, which is the expected result, because this dataset consists of gestures performed by six different users (Table 1). The *RMSE* for *GV*_3_ appears to have low *intraclass* variation, as expected, because the gestures are performed by an expert, who is able to repeat them in a very precise way.

**Table 1:** RMSE between the iterations of the real data of *GV*_2_ and *GV*_3_.

***GV*_2_**	$\bar{G_{2,1}}$	$\bar{G_{2,2}}$	$\bar{G_{2,3}}$	$\bar{G_{2,4}}$	$\bar{G_{2,5}}$
**RMSE**	0.0565	0.0523	0.0556	0.0330	0.0407
***GV*_3_**	$\bar{G_{3,1}}$	$\bar{G_{3,2}}$	$\bar{G_{3,3}}$	$\bar{G_{3,4}}$	
**RMSE**	0.0265	0.0302	0.0489	0.0461	

Table 2Gesture vocabulary of TV assembling dataset, AGV commands dataset, Glassblowing and Human-Robot Collaboration dataset, respectively.

**Table:** 

***GV***_**1**_ **-TV assembly**			
*G*_1,1_: Take the card from the left side box	*G*_1,2_: Take the wire from the right-side box	*G*_1,3_: Connect the wire with the card	*G*_1,4_: Place the card on the TV chassis
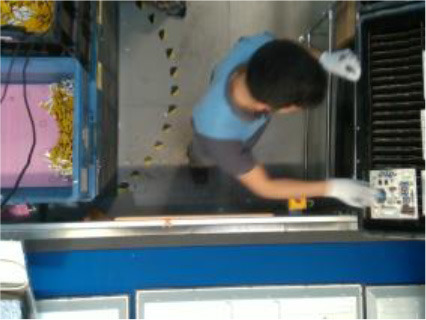	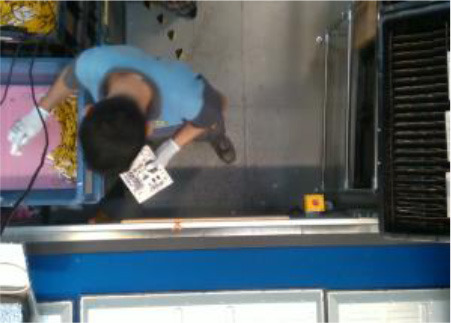	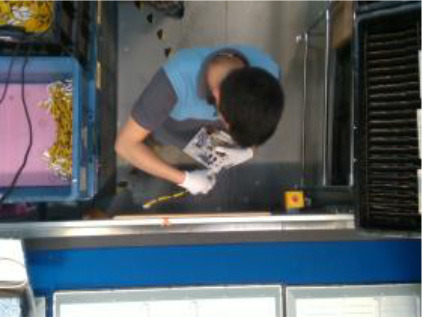	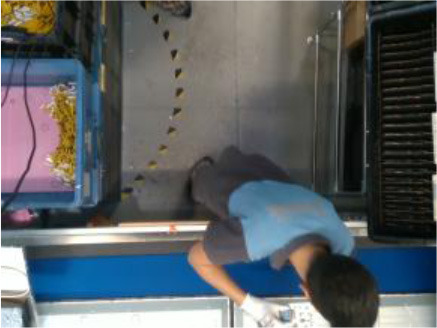

**Table:** 

***GV***_**2**_**-AGV commands**				
*G*_2,1_: Hello	*G*_2,2_: Left	*G*_2,3_: Right	*G*_2,4_: Speed up	*G*_2,5_: Speed down
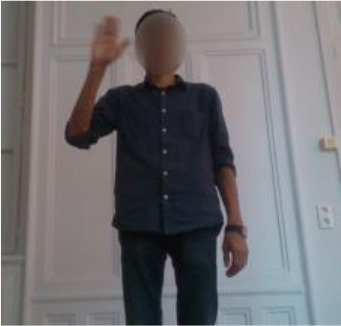	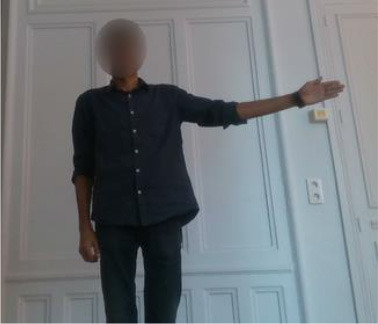	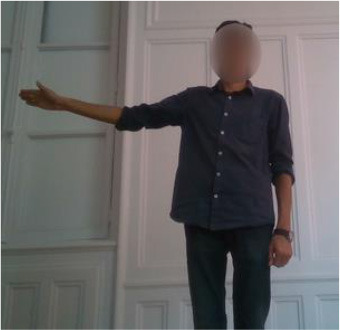	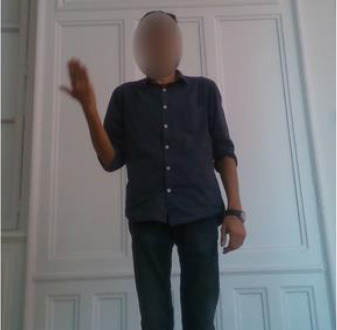	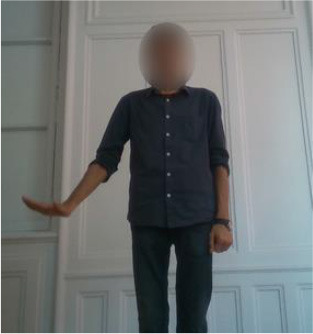

**Table:** 

***GV***_**3**_**-Glassblowing**			
*G*_3,1_: Fix details with pliers	*G*_3,2_: Tighten base of glass	*G*_3,3_: Make shape with paper	*G*_3,4_: Fix shape
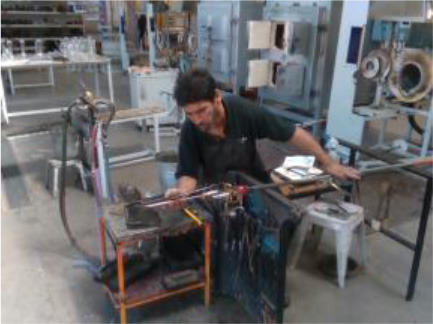	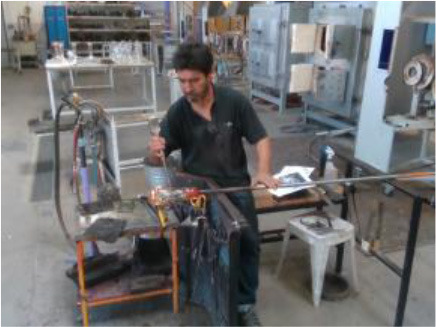	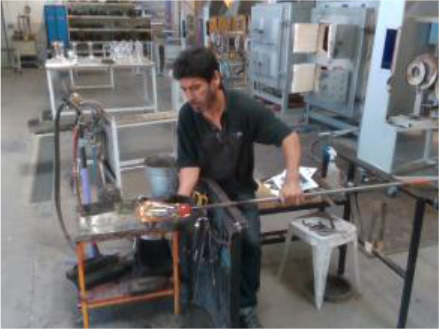	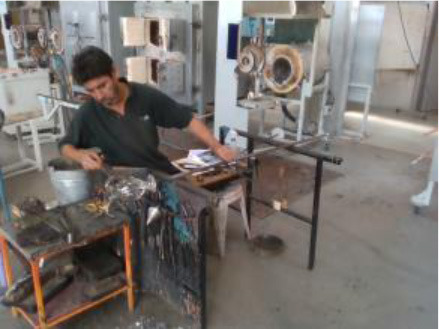

**Table:** 

***GV***_**4**_**-Human-robot collaboration**				
*G*_4,1_: Take a motor hose part in the robot right claw	*G*_4,2_: Take a motor hose part in the robot left claw	*G*_4,3_: Join two parts of the motor hose	*G*_4,4_: Screw	*G*_4,5_: Put the final motor hose in a box
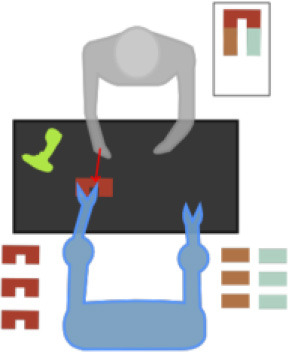	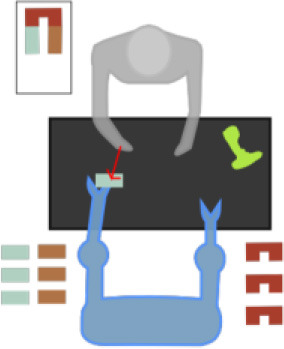	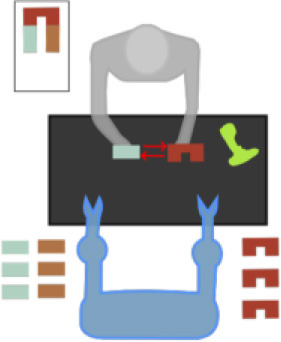	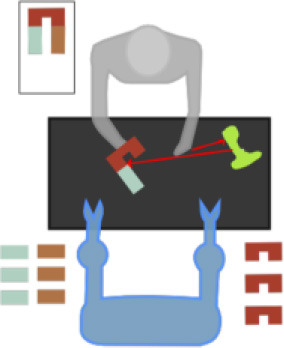	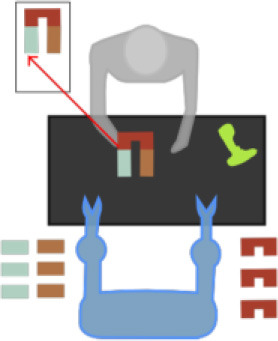

### Pose Estimation and Feature Extraction

After the motion capturing and recording of the data, each image sequence of the three first datasets is imported to the OpenPose framework, which detects body keypoints on the RGB image and extracts a skeletal model together with the 2D positions of each body joint (Cao et al., 2019) (Figure 2). These joints are not necessarily physical joints. They are keypoints on the RGB image, which, in most cases, correspond to physical joint centers. OpenPose uses the neck as the root keypoint to compute all the other body keypoints (or joints). Thus, the motion data are normalized by using the neck as the reference joint. In addition to this, the coordinates of each joint are derived by the width and height of the camera. With regard to *GV*_4_, 3D hand positions are extracted from top-mounted depth imaging by detecting keypoints on the depth map. The keypoints are localized by computing the geodesic distances between the closest body part to the camera (head) and the farthest visible body part (hands), as it is presented in our previous work (Manitsaris et al., 2015a). Any vision-based pose estimation framework may output 2D positions of a low precision, depending on the location of the camera, such as OpenPose for a top-mounted view. However, these errors may not strongly affect the recognition accuracy of our hybrid approach. This is also proved by the fact that our approach outperforms the end-to-end 3DCNNs that does not use any skeletization of the human body to recognize the human actions.

The extracted features for each joint, were the *X* and *Y* positions, as they are provided by OpenPose. More specifically, for *GV*_1_, the 2D positions of the two wrists have been used, whereas for *GV*_2_ and *GV*_3_, the 2D positions of the head, neck and shoulder, elbows, wrists, and hands have been used, as they were proven to give optimal recognition results. With regard to *GV*_4_, 3D hand positions are used.

### Gesture Operational Model

When a skilled individual performs a professional situated gesture, the whole body is involved combining, thus, theoretical knowledge with practical motor skills. Effective and accompanying body movements are harmonically coordinated to execute a given action. The expertise in the execution of professional gestures is characterized by precision and repeatability, while the body is continuously shifting from one phase to another, for example, from specific postures (small tolerance for spatial variance) to ample movements (high tolerance for spatial variance). For each phase of the movement, each body entity, for example, articulation or segment, moves in a multidimensional space over time. When considering the 2D motion descriptors of the movement, two mutually dependent variables represent the entity, for example, *X* and *Y* positions. Each of these variables is associated with the other, creating thus a bidirectional relationship between them. Furthermore, they also depend on their history, whereas some entities might “work together” to execute an effective gesture, for example, when an operator assembles two parts. However, a unidirectional dependency might be observed when one entity influences the other entity and not vice versa as well as a bidirectional dependency when both entities influence one each other, for example, when a potter shapes the clay with both hands.

The above observations on situated body movements can be translated into a functional model, which we define here as the GOM, which describes how the body skeletal entities of a skilled individual are organized to deliver a specific result (Figure 2). It is assumed that each of the assumptions of “intrajoint association,” “transitioning,” “intralimb synergies,” and “intralimb mediation” contribute at a certain level to the production of the gesture. As far as the *intralimb mediation* is concerned, it can be decomposed into the “interjoint serial mediation” and the “interjoint non-serial mediation.” The proposed model works perfectly for all three dimensions (*X*, *Y*, and *Z*), but for reasons of simplicity, it will be presented only for two dimensions, the *X* and *Y*. In addition to this, in this work, only positions are used, but the model is designed to be able to receive joint angles as input as well.

#### Intrajoint Association

It is hypothesized that the motion of each body part (*Entity*) (e.g., right hand) is decomposed in a motion on the *X*-axis and *Y*-axis, thus described by two mutually dependent variables. It is assumed that there is a bidirectional relationship between the two variables, defined here as *intrajoint association* and indicated by 
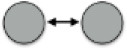
.

#### Transitioning

It is also assumed that each variable depends on its own history, also called inertia effect. This means that the current value of each variable depends on the values of previous times, also called lag or dynamic effect, which is defined here as *transitioning* and indicated by 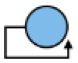.

#### Interlimb Synergies

It is assumed that some body entities work together to achieve certain motion trajectories, for example, hands when assembling two parts, defined here as *interlimb synergies*.

#### Intralimb Mediation

##### Interjoint serial mediation

It is assumed that a body entity may depend on its neighboring entities to which it is directly connected to; for example, a glassblower, while using the pipe, moves his/her wrists along with his/her shoulders and elbows. In case this assumption is statistically significant, there is an *interjoint serial mediation*.

##### Interjoint non-serial mediation

It is assumed that each body entity depends on non-neighboring entities of the same limb; for example, the movement of the wrist may depend on the movement of the elbow and shoulder. Thus, it is highly likely that both direct and indirect dependencies simultaneously occur in the same gesture. Entities are named after the first letters of the respective body joint. More specifically, LSH and RSH represent the left and right shoulders, respectively. Accordingly, LELBOW and RELBOW represent the left and right elbows; LWRIST and RWRIST, the left and right wrists; and LHAND and RHAND, the left and right hands. HEAD, NECK, and HIPS represent, as their names indicate, the head, the neck, and the hips.

So an example of the representation of those assumptions for the *X*-axis would be as follows:

(2) $$\begin{array}{l} Entity_{1,X}\left(t\right)=Entity_{1,Y}\left(t-1\right)+Entity_{1,X}\left(t-1\right)+\\ \;+Entity_{1,X}\left(t-2\right)+Entity_{2,X}\left(t-1\right)\\ \;+Entity_{3,X}\left(t-1\right) \end{array}$$

### Simultaneous Equation System

The simultaneous equation system concatenates the dynamics of an Nth-order system, the GOM, into *N* first-order differential equations. The number of equations is equal to the number of associated dimensions to a given entity multiplied by the number of body entities. Therefore, the steps to follow are the estimation of the model, with the aim of verifying its structure, as well as the simulation of the model to verify its forecasting ability.

#### State-Space Representation

The definition of the equations of the system follows the theory of the SS modeling, which gives the possibility for the coefficients to dynamically change over time. An SS model for *n*-dimensional time series *y*(*t*) consists of a *measurement or observation equation* relating the observed data to an *m*-dimensional state vector *s*(*t*) and a Markovian *state or transition equation* that describes the evolution of the state vector over time. The *state equation* depicts the dependence between the system's past and future and must “canalize” through the state vector. The *measurement or observation equation* is the “lens” (signal) through which the hidden state is observed, and it shows the relationship between the system's state, input, and output variables. Representing a dynamic system in an SS form allows the state variables to be incorporated into and estimated along with the observable model.

Therefore, given an input *u*(*t*) and a state *s*_*S*_(*t*), a SS gives the hidden states that result to an observable output (signal). A general SS representation is as follows:

(3) $$\begin{array}{l} \frac{ds_{s}}{dt}=As_{S}\left(t-1\right)+w\left(t\right) \end{array}$$

(4) $$\begin{array}{l} y=C\frac{ds_{s}}{dt}+Du \end{array}$$

where Equation (3) is the *state* equation, which is a first-order Markov process; Equation (4) is the *measurement* equation; *s*_*S*_ is the vector of all the state variables; $\frac{ds_{s}}{dt}\;$is the time derivative of the state vector; *u* is the input vector; *y* is the output vector; *A* is the transition matrix that defines the weight of the precedent space; *C* is the output matrix; and *D* is the feed-through matrix that describes the direct coupling between *u* and *y*; and *t* indicates time.

When capturing the gestures with motion sensors, Gaussian disturbances (w(t)) are also added in both the state and output equations. After the experiments presented in this work were performed, it was observed that Gaussian disturbances did not change at all the final estimation result, so they were considered to be negligible.

The SS representation of the positions on the *X*-axis for a body *Entity*_*i,j*_, *where* *i* *represents the body part modeled in an SS form and* *j* *the dimension of each* *Entity*- according to the GOM structured, as follows:

(5) $$\begin{array}{l} \frac{ds_{s}}{dt}=A*s_{S}\left(t-1\right)=\left[\begin{array}{c} \alpha_{1}\;0\\ 0\;\alpha_{2} \end{array}\right]\left[\begin{array}{c} Entity_{1,X}\left(t-1\right)\\ -Entity_{1,X}\left(t-2\right) \end{array}\right]\\ \;=\left[\begin{array}{c} \alpha_{1}Entity_{1,X}\left(t-1\right)\\ -\alpha_{2}Entity_{1,X}\left(t-2\right) \end{array}\right] \end{array}$$

(6) $$\begin{array}{l} \overset{\left(5\right)}{\Rightarrow }Entity_{1,X}\left(t\right)=\left[1\;1\right]\frac{ds_{s}}{dt}+\alpha_{3}Entity_{1,Y}(t-1)+\\ \;+\alpha_{4}Entity_{2,X}\left(t-1\right)\\ \;=\alpha_{1}Entity_{1,X}\left(t-1\right)-\alpha_{2}Entity_{1,X}(t-2)+\\ \;+\alpha_{3}Entity_{1,Y}\left(t-1\right)+\alpha_{4}Entity_{2,X}\left(t-1\right) \end{array}$$

where α_*i*_ are the coefficients that need to be estimated. For simplicity, the inter-joint non-serial mediation is not used in the specific example. In Equation (6), *Entity*_*X*_(*t* − 2) is subtracted by *Entity*_*X*_(*t* − 1), indicating the difference between successive levels of dimensions, for example, positions on the *Y*-axis (transitioning assumption). Equations (5) and (6) occur by Equations (3) and (4), respectively. More specifically, Equation (6) consists of the exogenous variables to which the endogenous ones, coming up from the state equation (Equation 5), are added.

Equation (6) has now the form of a second-order autoregressive (AR) model. An AR model predicts future behavior on the basis of past behavior. The order of the AR model is adapted in each case according to the data characteristics and the experiments. During the performance of the experiments, the use of an AR model of second order led to better estimation results. As such, in the transitioning assumption, the position values of the two previous time periods (frames) of a given axis are considered.

For the modeling of the full human body, the simultaneous equation system is based on Equations (5) and (6), which consists of two sets of equations for each used entity, one for each dimension *X* and *Y*. Thus, for a full-body GOM, we obtain 32 equations describing 32 variables with 64 state variables that contain two endogenous variables for *t* − 1 and *t* − 2.

As an example, the SS representation for the right wrist is given as follows:

(7) $$\begin{array}{l} RWRIST_{X}\left(t\right)=\alpha_{1}RWRIST_{X}\left(t-1\right)-\alpha_{2}RWRIST_{X}\left(t-2\right)+\\ \;+\alpha_{3}RWRIST_{Y}\left(t-1\right)+\alpha_{4}LWRIST_{X}\left(t-1\right) \end{array}$$

In Equation (7), *RWRIST*_*X*_(*t* − 1) and *RWRIST*_*X*_(*t* − 2) are the endogenous variables, whereas *RWRIST*_*Y*_(*t* − 1), and *LWRIST*_*X*_(*t* − 1) are the exogenous ones.

### Computing the Coefficients of the Equations

The coefficients of the simultaneous equation system are computed using the MLE method via Kalman filtering (Holmes, 2016). Let us consider a gesture *G*_*j*∈ℕ_ of a gesture vocabulary *GV*_*i*∈[1, 3]_ and an observation $O_{0:k}={\left\{o_{1},\ldots {,o}_{k}\;\right\}}_{k\in \mathbb{N}}$, where *o*_*k*_ is one observation vector and *k* the total number of observations. Thus, the probability $P_{s}$ to observe *o*_*t*_ at time *t* ∈ [0, *k*] will be as follows:

(8) $$\begin{array}{l} P_{s}\left(O_{0:k}\right)=\overset{k}{\underset{t=0}{\prod }}P\left(o_{t}\;|O_{0:t-1}\right) \end{array}$$

where *k* represents the observed data, $P\left(o_{t}|O_{0:t-1}\right)$ is the probability of *o*_*t*_ given all the observations before time *t*.

Also, the probability of time series given a set of parameters Ψ is

(9) $$P\left(O_{0:t-1}|\Psi \right)=\prod_{t=1}^{k}\exp\left\{-\frac{{\left(o_{t}-{\tilde{o}}_{t}^{t-1}\right)}^{2}}{2F_{t}^{t-1}}\right\}{\left(2\pi |F_{t}^{t-1}|\right)}^{-\frac{1}{2}}d\theta$$

with variance $F_{t}^{t-1}\;$and mean ${õ}_{t}^{t-1}.\;$So the log-likelihood of ψ given data $O_{0:t-1}$ is

(10) $$\begin{array}{l} \mathrm{logL}\left(\Psi |O_{0:t-1}\right)=-\frac{k}{2}\log2\pi -\frac{1}{2}\overset{k}{\underset{t=1}{\sum }}\log|F_{t}^{t-1}|-\\ \;-\frac{1}{2}\overset{k}{\underset{t=1}{\sum }}\frac{{\left(o_{t}-{õ}_{t}^{t-1}\right)}^{2}}{F_{t}^{t-1}} \end{array}$$

For the computation of this log-likelihood, the estimation, the variance, and mean that appear in Equation (4) need to be estimated optimally. Kalman filtering gives the optimal estimates of the mean and covariance for the calculation of the maximum likelihood of ψ. Kalman filtering consists of two main recursive steps, prediction and update. In the first step, there is an estimation of the mean and covariance, along with the predicted error covariance. In the update step, the optimal Kalman gain is computed, so the estimation of mean and covariance from the prediction step is updated according to it. These two steps appear recursively, until the optimal ${õ}_{t}^{t-1}$ and $F_{t}^{t-1}$ that fit the observed data are computed. This derives the computation of the coefficients of the SS equations and the forecasting of a new time series given those observed data.

### Learning With Hidden Markov Models

HMMs follow the principles of Markov chains that describe stochastic processes. They are commonly used to model and recognize human gestures. They are structured using two different types of probabilities, the transition probability from one state to another and the probability for a state to generate specific observations on the signal (Bakis, 1976). In our case, each professional gesture is associated to an HMM, whereas the intermediate phases of the gesture constitute internal states of the HMM. According to our datasets, these gestures define the gesture vocabulary *GV*_*i*∈[1, 3]_ = {*G*_*j*_}_*j*∈ℕ_.

Let $S_{h}$ be a finite space of states, corresponding to all the intermediate phases of a professional gesture. The transition probability is between the states $Q\;\left(s_{h},\;s_{h}^{\prime }\right)$, where $s_{h},\;s_{h}^{\prime }\in S_{h}$ are given in the transition matrix $Q=\left[Q\left(s_{h},\;s_{h}^{\prime }\right)\right]$. A hidden sequence of states where $S_{h0:k}={\left\{s_{h1},\ldots ,s_{hk}\;\right\}}_{k\in \mathbb{N}}$, where $s_{hk}\in S_{h}$ is also considered. A given sequence of hidden states *S*_*h*0:*k*_ is supposed to generate a sequence of observation vectors $O_{o:k}$. We assume that the vectors *o*_*k*_ depends only on the state *s*_*hk*_. From now on, the likelihood that the observation *o* is the result of the state *s*_*h*_ will be defined as $P_{h}\left(o|s_{h}\right)$. It is important to outline that in our modeling structure, each internal state of the model depends only on its previous state (first-order Markov property). Consequently, the set of the models for all gestures for every gesture vocabulary is *GV*_*j*∈[1, 3]_ = {*HMM*_*i*_}_*i*∈ℕ_, where $HMM_{i}={\left(\varrho_{i},Q_{i},P_{hi}\right)}_{i\in \mathbb{N}}$ are the parameters of the model and ϱ_*i*_ is the initial state probability. Thus, the recognition becomes an issue of solving three specific problems: *evaluation, recognition*, and *learning* (Dymarski, 2011). Each one of those problems was solved with the use of the algorithms, Viterbi (Rabiner, 1989), Baum's “forward” (Baum, 1972), and Baum–Welch, respectively (Dempster et al., 1977).

### Gesture Recognition

In the recognition phase, the main goal is to recall with a high precision the hidden sequence of internal states $S_{h0:k}$ that correspond to the sequences of the observation vectors. Thus, let us consider the observation of motion data $O_{0:k}$, which need to be recognized. Every *HMM*_**λ**_ with λ ∈ [1, *j*] of a given *GV*_*i*_ with *i* ∈ [1, 3] generates the likelihood $P_{h\text{λ}}(O_{0:k}|HMM_{\text{λ}})$. If there is at least one *HMM*_ξ_ with ξ ∈ [1, *j*] that generates $P_{h\xi }\geq 0.55$, then it is considered that $O_{0:k}$ is generated by *G*_*i*, ξ_. Otherwise, the following quantity is computed for every *SS*_λ_ of *GV*_*i*_ (confidence control):

(11) $$\begin{array}{l} SS_{\lambda }^{score}=\frac{1}{1+d\left(O_{0:k},O_{0:k,\lambda }^{s}\right)} \end{array}$$

where *d* is the minimum distance between the simulated values $O_{0:k,\lambda }^{s}$ from the model *SS*_λ_ and the original observations $O_{0:k}$.

Then, for every *SS*_λ_ of *GV*_*i*_, the likelihood $P\prime_{h\text{λ}}\left(O_{0:k}|HMM_{\text{λ}}^{SS}\right)$ is computed as follows:

(12) $$P\prime_{h\lambda }\left(O_{0:k}|HMM_{\lambda }^{SS}\right)=P_{h\lambda }(O_{0:k}|HMM_{\lambda })\cdot SS_{\lambda }^{score}$$

Therefore, the final formula provides the way the algorithm recognizes the observation of motion data $O_{0:k}$,

(13) $$\begin{array}{l} R_{GV_{i}}\left(O_{0:k}\right)=\;\{\begin{array}{c} \;\max_{1}^{j}\left(P_{hi}(O_{0:k}|HMM_{i})\right),\;\max\left(P_{h\lambda }(O_{0:k}|HMM_{\lambda })\right)\\ \geq 0.55\;\\ \max_{1}^{j}\left(P\prime_{h\lambda }\left(O_{0:k}|HMM_{\lambda }^{SS}\right)\right),\;\max\left(P_{h\lambda }(O_{0:k}|HMM_{\lambda })\right)\\ <0.55\; \end{array} \end{array}$$

## Evaluation

The evaluation of the accuracy and performance of the method follows an “all-shots” approach for the training of the HMMs and a “one-shot” approach for estimating the coefficients of the SS models.

In order to select which gestural iteration to use for computing the coefficients of the SS models, the “leave-one-out method” is used. It is a resampling technique that is also useful for variance and bias estimation (and avoidance), especially when the data are limited. It consists in systematically leaving out one observation from a dataset, calculating the estimator, and then finding the average of these calculations. In our case, the estimator was the likelihood of the HMM when trained with one iteration of a gesture and tested with all the other iterations. The iteration giving the maximum likelihood is selected for computing the coefficients of the SS models.

## Statistical Significance and Simulation of the Models

In order to evaluate the significance of the assumptions concerning the body part dependencies that are defined within the GOM, a statistical significance analysis is done. The statistical significance *p*-value indicates whether the assumptions are verified or not. The level of statistical significance is often expressed by using the *p*-value, which takes values between 0 and 1. Generally, the smaller the *p*-value, the stronger the evidence that the null hypothesis should be rejected. In this work, the 0.05 *p*-value was used as the threshold for the statistical significance tests. If the *p*-value of the estimated coefficient is smaller than 0.05, then the specific coefficient is statistically significant and need to be included in the SS representation of the model.

In the case of the professional gestures, investigating the significance level of the coefficients of each variable within the GOM explains how important is each joint for each gesture in the gestural vocabulary. Examples of some of the gestures from *GV*_2_ and *GV*_3_ are given, to observe cases where some of the coefficients affect strongly the results and need to remain dynamic, whereas others cannot, and can remain constant. In the GOM below, the equation of *G*_2,1_ for *RWRIST*_*X*_ is as follows, starting from Equation (2).

(14) $$\begin{array}{l} RWRIST_{X}\left(t\right)=a_{12}RWRIST_{Y}\left(t-1\right)+a_{13}RWRIST_{X}\left(t-1\right)-\\ \;-a_{14}RWRIST_{X}\left(t-2\right)+a_{15}LWRIST_{X}\left(t-1\right)\\ \;=\overset{\;0.266}{\overset{︷}{-0.0629}}\;RWRIST_{Y}\left(t-1\right)+\\ \;+\overset{0.00}{\overset{︷}{1.3438}}RWRIST_{X}\left(t-1\right)-\\ \;-\left(\overset{0.00}{\overset{︷}{-0.3648}}\right)RWRIST_{X}\left(t-2\right)+\\ \;+\left(\overset{0.449}{\overset{︷}{-0.6625}}\right)LWRIST_{X}\left(t-1\right) \end{array}$$

Having performed the statistical significance analysis of the model in Equation (14), we get the estimation of the coefficients, where Equation (14) is the general equation for *X*-axis of the right wrist, along the *p*-values that indicate the level of significance of each part of the equation. The *p*-values show that in the case of the *G*_2,1_, the past values on the same axis appear to be significant, whereas the respective *p*-values of the left wrist or the Y-axis of the right wrist are not statistically significant. This result was expected, as this gesture is a “hello waving movement,” where the right wrist is moving across the *X* axis and the left wrist remains still through the performance of the gesture, leading to the result that there is no intralimb mediation in this specific gesture.

In the following, there is one more example of the same *GV*, from gesture *G*_2,3_, for *X*-axis (Equation 15) and *Y*-axis (Equation 16). The numbers above the estimated coefficients correspond to their respective *p*-values.

(15) $$\begin{array}{l} RWRIST_{X}\left(t\right)=a_{12}RWRIST_{Y}\left(t-1\right)+a_{13}RWRIST_{X}\left(t-1\right)-\\ \;-a_{14}RWRIST_{X}\left(t-2\right)+a_{15}LWRIST_{X}\left(t-1\right)\\ \;=\overset{0.00}{\overset{︷}{-0.2871}}\;RWRIST_{Y}\left(t-1\right)+\overset{0.00}{\overset{︷}{0.6392}}\times \\ \;\times RWRIST_{X}\left(t-1\right)\overset{0.86}{\overset{︷}{-0.0273}}RWRIST_{X}\left(t-2\right)+\\ \;+\overset{0.00}{\overset{︷}{0.0516}}\;LWRIST_{X}\left(t-1\right) \end{array}$$

(16) $$\begin{array}{l} RWRIST_{Y}\left(t\right)=a_{12}RWRIST_{X}\left(t-1\right)+a_{13}RWRIST_{Y}\left(t-1\right)-\\ \;-a_{14}RWRIST_{Y}\left(t-2\right)+a_{15}LWRIST_{Y}\left(t-1\right)\\ \;=\overset{0.00}{\overset{︷}{-3.9907}}\;RWRIST_{X}\left(t-1\right)+\\ \;+\overset{0.00}{\overset{︷}{0.5003}}\;RWRIST_{Y}\left(t-1\right)-\\ \;-{\overset{0.616}{\overset{︷}{\left(-0.0818\right)}}RWRIST}_{Y}\left(t-2\right)+\\ \;+{\overset{0.00}{\overset{︷}{\left(-0.0927\right)}}LWRIST}_{Y}\left(t-1\right) \end{array}$$

In this gesture, the operator moves his/her right wrist toward his/her right side both on the *X* and *Y* axes, indicating to the AGV to turn right. So according to the results, all coefficients appear to be statistically significant, apart from the two previous time-period values of the *X*-axis of the right wrist. The same results occur for the Y-axis of the same wrist.

To verify the results, a significance level test is presented for *G*_3,2_ of *GV*_3_. During the performance of this gesture, the glassblower is moving both wrists cooperatively, to tighten the base of the glass piece. The right wrist works more intensively to complete tightening the glass, whereas the left wrist complements the movement by slowly rolling the metal pipe.

(17) $$\begin{array}{l} {RWRIST}_{X}\left(t\right)=a_{12}RSH_{X}\left(t-1\right)+a_{13}RELBOW_{X}\left(t-1\right)+\\ \;+a_{14}RWRIST_{Y}\left(t-1\right)+a_{15}LWRIST_{X}\left(t-1\right)+\\ \;+a_{16}\;RWRIST_{X}\left(t-1\right)-a_{17}RWRIST_{X}\left(t-2\right)\\ \;=\left(\overset{0.562}{\overset{︷}{-0.0778}}\right)RSH_{X}\left(t-1\right)+\overset{0.00}{\overset{︷}{1.1126}}\;RELBOW_{X}\left(t-1\right)+\\ \;+\left(\overset{0.00}{\overset{︷}{-0.4757}}\right)\;RWRIST_{Y}\left(t-1\right)+\overset{0.00}{\overset{︷}{0.3423}}\times \\ \;\times LWRIST_{X}\left(t-1\right)+\overset{0.00}{\overset{︷}{0.4585}}\;RWRIST_{X}\left(t-1\right)+\\ \;-\overset{0.00}{\overset{︷}{0.4604}}\;RWRIST_{X}\left(t-2\right) \end{array}$$

(18) $$\begin{array}{l} RWRIST_{Y}\left(t\right)=a_{12}RSH_{Y}\left(t-1\right)+a_{13}RELBOW_{Y}\left(t-1\right)+\\ \;+a_{14}RWRIST_{X}\left(t-1\right)+a_{15}LWRIST_{Y}\left(t-1\right)+\\ \;+a_{16}\;RWRIST_{Y}\left(t-1\right)-a_{17}RWRIST_{Y}\left(t-2\right)\\ \;=\overset{0.117}{\overset{︷}{0.290}}\;RSH_{Y}\left(t-1\right)+\overset{0.00}{\overset{︷}{0.3678}}RELBOW_{Y}\left(t-1\right)+\\ \;+\left(\overset{0.00}{\overset{︷}{-1.0912}}\right)\;RWRIST_{X}\left(t-1\right)+\left(\overset{0.045}{\overset{︷}{-0.1602}}\right)\times \\ \;\times LWRIST_{Y}\left(t-1\right)+{\overset{0.00}{\overset{︷}{1.1298}}\;RWRIST}_{Y}\left(t-1\right)+\\ \;-\left(\overset{0.00}{\overset{︷}{-0.1679}}\right)\;RWRIST_{Y}\left(t-2\right) \end{array}$$

(19) $$\begin{array}{l} LWRIST_{X}\left(t\right)=a_{12}LSH_{X}\left(t-1\right)+a_{13}LELBOW_{X}\left(t-1\right)+\\ \;+a_{14}\;LWRIST_{Y}\left(t-1\right)+a_{15}RWRIST_{X}\left(t-1\right)+\\ \;+a_{16}\;LWRIST_{X}\left(t-1\right)-a_{17}LWRIST_{X}\left(t-2\right)\\ \;=\overset{0.00}{\overset{︷}{0.3668}}\;LSH_{X}\left(t-1\right)+\overset{0.007}{\overset{︷}{0.11180}}\;LELBOW_{X}\left(t-1\right)+\\ \;+\overset{0.00}{\overset{︷}{0.9589}}\;LWRIST_{Y}\left(t-1\right)+\left(\overset{0.339}{\overset{︷}{-0.0126}}\right)\times \\ \;\times RWRIST_{X}\left(t-1\right)+{\overset{0.00}{\overset{︷}{1.1111}}LWRIST}_{X}\left(t-1\right)+\\ \;-\left(\overset{0.052}{\overset{︷}{-0.1398}}\right)\;LWRIST_{X}\left(t-2\right) \end{array}$$

(20) $$\begin{array}{l} LWRIST_{Y}\left(t\right)=a_{12}LSH_{Y}\left(t-1\right)+a_{13}LELBOW_{Y}\left(t-1\right)+\\ \;+a_{14}LWRIST_{X}\left(t-1\right)+a_{15}RWRIST_{Y}\left(t-1\right)+\\ \;+a_{16}LWRIST_{Y}\left(t-1\right)-a_{17}LWRIST_{Y}\left(t-2\right)\\ \;=\overset{0.00}{\overset{︷}{0.9313}}\;LSH_{Y}\left(t-1\right)+\overset{0.00}{\overset{︷}{0.5433}}\;LELBOW_{Y}\left(t-1\right)\\ \;+\overset{0.272}{\overset{︷}{0.1144}}\;LWRIST_{X}\left(t-1\right)+\overset{0.356}{\overset{︷}{0.0162}}\;RWRIST_{Y}\left(t-1\right)+\\ \;+\overset{0.0}{\overset{︷}{1.0463}}\;LWRIST_{Y}\left(t-1\right)-\overset{0.124}{\overset{︷}{\left(-0.1095\right)}}\;LWRIST_{Y}\left(t-2\right) \end{array}$$

In the equations presented above, all coefficients appear to be statistically significant, except from *RSH*_*X*_(*t* − 1) in Equation (17), *LWRIST*_*X*_(*t* − 1) in Equation (18), *LWRIST*_*X*_(*t* − 1) and *RWRIST*_*X*_(*t* − 2) in Equation (19), and *RWRIST*_*X*_(*t* − 1), *RWRIST*_*Y*_(*t* − 1), *and LWRIST*_*X*_(*t* − 1) in Equation (20). As a result, the hands of the operator work mostly independently (there appears to be a dependency in the interlimb synergies in Equation 17), whereas all the other assumptions seem to be statistically significant for both *X*-axis and *Y*-axis of the right and left wrists.

**Figure 3:**
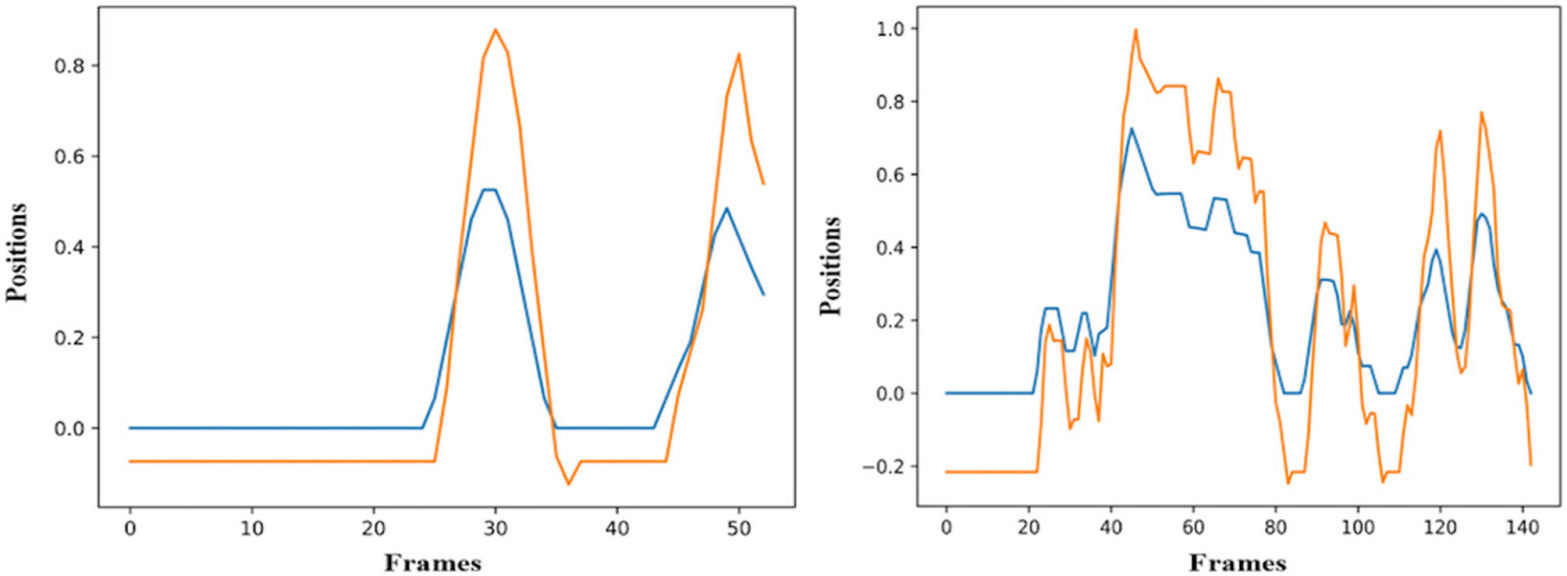
Examples of real motion observations (blue) and simulated values (orange) from the *RHAND*_*X*_ State-Space model of the gesture *G*_1,1_ (left) and *G*_1,4_ (right).

**Figure 4:**
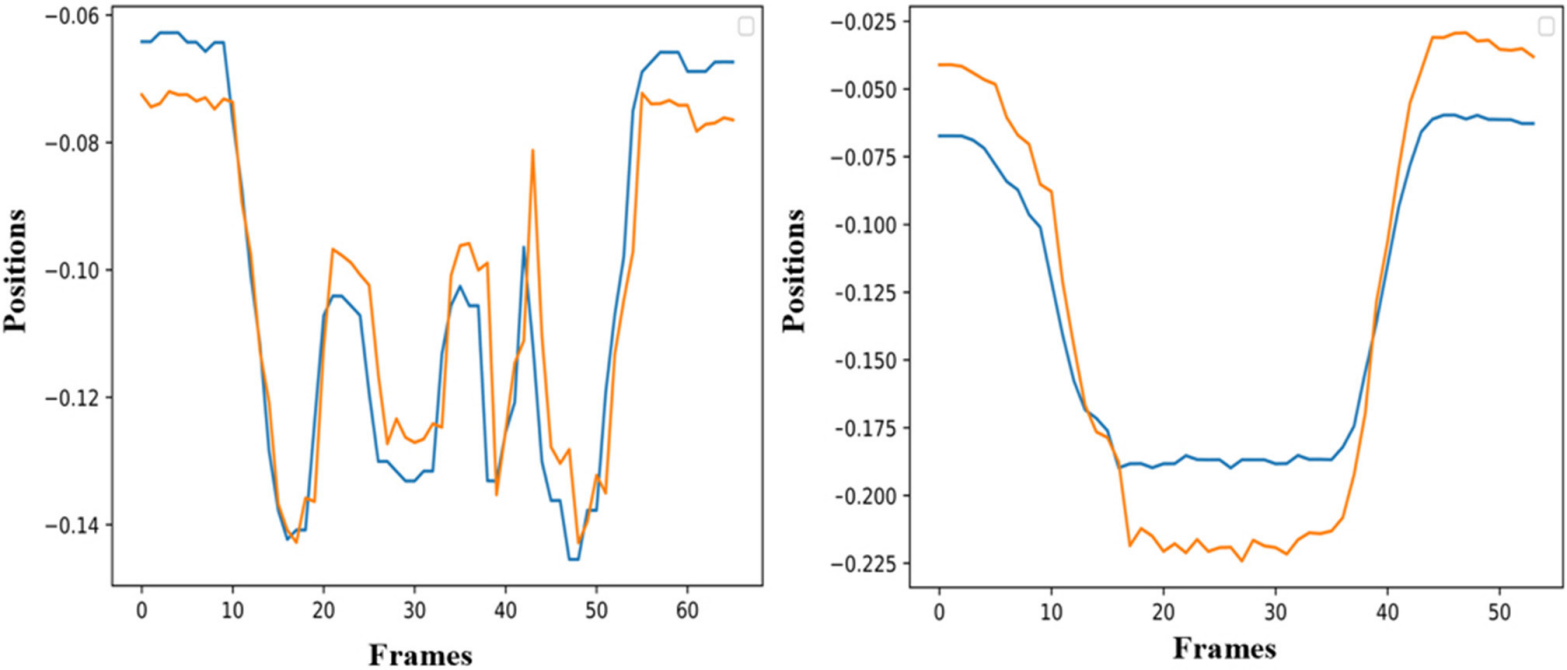
Examples of real motion observations of motion data (blue) and simulated values (orange) from the *RHAND*_*X*_ State-Space model of gesture *G*_2,1_ (left) and *G*_2,2_ (right).

**Figure 5:**
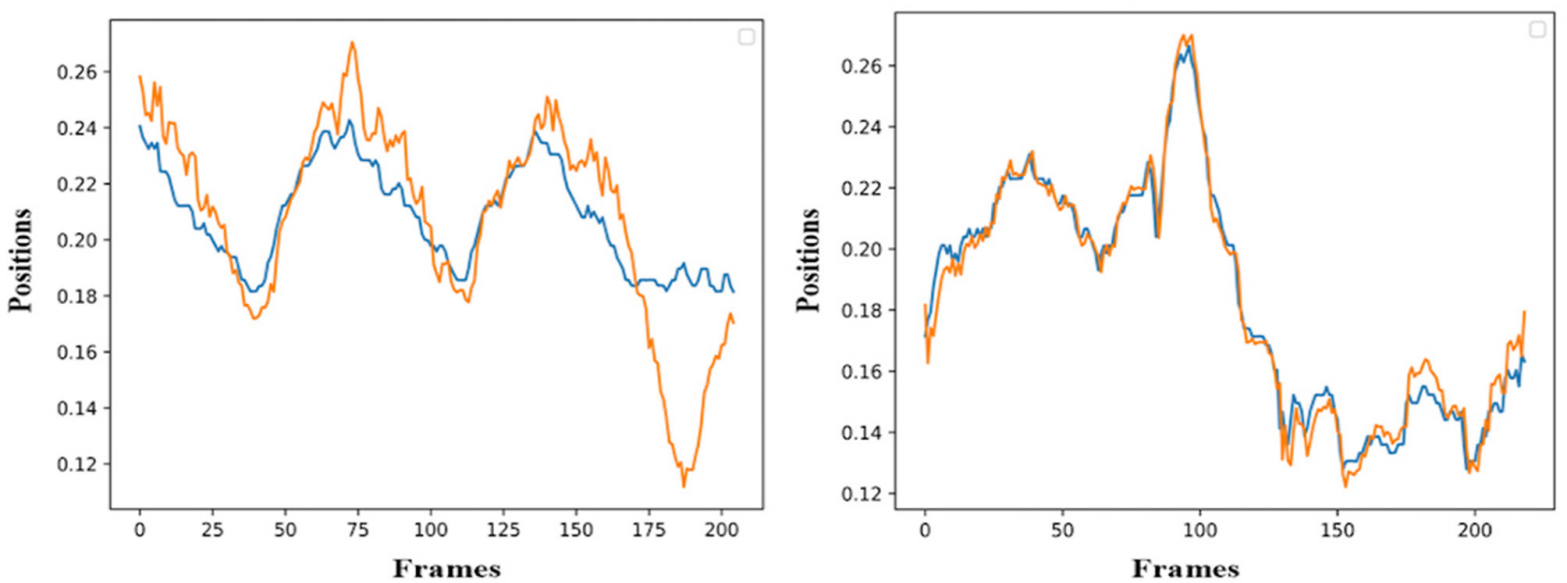
Examples of real motion observations (blue) and simulated values (orange) from the *RHAND*_*X*_ State-Space model of the gesture *G*_3,1_ (left) and *G*_3,4_ (right).

The simulation of the models is based on the solution of their simultaneous equations system. Figures 3–5 show examples of the graphical depiction of real observations of motion data together with their simulated values from the SS model of the right wrist. A general conclusion that can be exported by looking at the depictions is that the behavior of the models is very good because the two curves are really close in most cases.

### Recognition Accuracy and Comparison with End-to-End Deep Learning Architectures

For the evaluation of the performance and the proposed methodology, the metrics *precision*, *recall*, and *f-score* were calculated. Those metrics are defined as shown below.

(21) $$\begin{array}{l} precision=\;\frac{\#\left(true\;positives\right)}{\#\left(true\;positives\right)+\#\left(false\;positives\right)} \end{array}$$

(22) $$\begin{array}{l} recall=\;\frac{\#\left(true\;positives\right)}{\#\left(true\;positives\right)+\#\left(false\;negatives\right)} \end{array}$$

*Precision*, *recall*, and *f-score* are calculated for all the gestures that each gestural vocabulary consists of. For a gesture of class *i*, #(*true positives*) represent the number of gestures of class *i* that were recognized correctly, #(*false positives*) represent the number of gestures that did not belong in class *i*, and they were recognized from the algorithm as parts of class *i*. Finally, #(*false negatives*) represents the number of gestures belonging to class *i* that were not recognized as part of it.

More precisely, *precision* represents the rate of gestures that really belong in class *i*, among those who are recognized as class *i*, whereas *recall* represents the rate of iterations of gestures of class *i* that have been recognized as class *i*. A measure that combines both precision and recall is the *f*-score, which is given by Equation (23).

(23) $$\begin{array}{l} f-score=2\frac{precision*recall}{precision+recall} \end{array}$$

The performance of the algorithms was tested with the four different gestural vocabularies. As presented before, the *GV*_1_ contains four classes, from 44 to 48 repetitions for each. Four hidden states were used for HMM training. To simplify the evaluation task, a simplified GOM with only *X* and *Y* positions of two wrists are used for training and recognition. **Table 4** presents the results when only the HMMs are used for recognition without any confidence control and also the results with the confidence control provided by the simulation of the SS models.

It is possible to observe that HMMs provide a recall superior to 90% in three out of four gestures. The *G*_1,3_ presents the lowest recall of 81.81%, and this can be due to the fact that this is the most complex gesture, where both hands interact more than in the other three gestures. The lowest precision is detected for the *HMM*_1,4_. When the SS representation and confidence control is used, the *recall* for *G*_1,2_ is slightly improved, whereas in the case of the *G*_1,3_, a significant improvement of 15.91% is achieved. Especially for *G*_1,3_, the improvement can be justified by the fact that the operator is connecting the wire with a very small card outside the conveyor. Thus, the operator has the possibility to perform very small movements in different positions of his/her workplace. The *precision* of $HMM_{1,4}^{SS}$ has been also positively impacted by the SS augmenting the precision from 90.2 to 97.67%. However, a slight decline can also be seen in the case of *G*_1,4_ recall (Table 3).

**Table 3:** Confusion matrix using *HMM, HMM*^*SS*^, and 3*DCNN* based on the model of Tran et al. (2015) approaches for *GV*_1_.

	*HMM*_1,1_	*HMM*_1,2_	*HMM*_1,3_	*HMM*_1,4_	**Recall (%)**
*G*_1,1_	48	0	0	0	100
*G*_1,2_	0	44	0	2	95.65
*G*_1,3_	1	4	36	3	81.81
*G*_1,4_	0	0	0	46	100
**Precision (%)**	97.9	91.66	100	90.2	
	${HMM}_{1,1}^{SS}$	${HMM}_{1,2}^{SS}$	${HMM}_{1,3}^{SS}$	${HMM}_{1,4}^{SS}$	**Recall (%)**
*G*_1,1_	47	0	0	1	97.91
*G*_1,2_	1	45	0	0	97.82
*G*_1,3_	0	1	43	0	97.72
*G*_1,4_	0	4	0	42	91.3
**Precision (%)**	97.91	90	100	97.67	
	3*DCNN*_1,1_	3*DCNN*_1,2_	3*DCNN*_1,3_	3*DCNN*_1,4_	**Recall (%)**
*G*_1,1_	48	0	0	0	100
*G*_1,2_	0	43	0	5	89.5
*G*_1,3_	0	0	44	0	100
*G*_1,4_	0	7	0	39	84.7
**Precision (%)**	100	86	100	88.6	

The *GV*_2_ contains five classes and 16 repetitions of each gesture, and one to 11 hidden states were used for the machine learning gesture recognition engine according to the best states' number for each iteration. The joints selected for training with *GV*_2_ were the wrist, elbow, and shoulder joints for each hand, along with the neck. In Table 4, *precision* and *recall* using only *HMM* and the *HMM*^*SS*^ approach is presented, respectively. For *G*_2,1_, *G*_2,4_, and *G*_2,5_, ergodic topology was used, as iterations of the same gestural part appear during the performance of each gesture, whereas left to right topology was used for the rest of the gestures. *Precision* appears improved for every model, whereas *recall* is decreased for *G*_2,2_ and *G*_2,5_ (Table 4).

**Table 4:** Confusion matrix using *HMM, HMM*^*SS*^, and 3*DCNN* based on the model of Tran et al. (2015) approach for *GV*_2_.

	*HMM*_2,1_	*HMM*_2,2_	*HMM*_2,3_	*HMM*_2,4_	*HMM*_2,5_	**Recall (%)**
*G*_2,1_	14	1	0	1	0	87.5
*G*_2,2_	3	13	0	0	0	81.25
*G*_2,3_	0	0	16	0	0	100
*G*_2,4_	5	0	0	11	0	68.75
*G*_2,5_	2	0	0	2	12	75
**Precision (%)**	58.3	92.8	100	78.5	100	
	${HMM}_{2,1}^{SS}$	${HMM}_{2,2}^{SS}$	${HMM}_{2,3}^{SS}$	${HMM}_{2,4}^{SS}$	${HMM}_{2,5}^{SS}$	**Recall (%)**
*G*_2,1_	16	0	0	0	0	100
*G*_2,2_	4	12	0	0	0	75
*G*_2,3_	0	0	16	0	0	100
*G*_2,4_	2	0	0	14	0	87.5
*G*_2,5_	3	0	0	3	10	62.5
**Precision (%)**	64	100	100	82.3	100	
	3*DCNN*_2,1_	3*DCNN*_2,2_	3*DCNN*_2,3_	3*DCNN*_2,4_	3*DCNN*_2,5_	**Recall (%)**
*G*_2,1_	16	0	0	0	0	100
*G*_2,2_	0	16	0	0	0	100
*G*_2,3_	0	0	16	0	0	100
*G*_2,4_	0	0	0	12	4	75
*G*_2,5_	8	0	0	0	8	50
**Precision (%)**	66.6	100	100	100	66.66	

*GV*_3_ consists of four different gestures with 35, 34, 21, and 27 repetitions, respectively. 5 to 20 hidden states were used for training the gesture recognition algorithm, the number of which were again computed for every iteration in the resampling phase. The joints selected for training with *GV*_3_ were again the wrist, elbow, and shoulder joints for each hand, along with the neck. *Precision* appears improved in almost every observation and maximum likelihood. The *recall* in almost every gesture has remained stable except from *G*_3,3_, where it was increased by +4% (Table 5).

**Table 5:** Confusion matrix using *HMM*, *HMM*^*SS*^, and the Tran et al. (2015) 3*DCNN* approach for *GV*_3_.

	*HMM*_3,1_	*HMM*_3,2_	*HMM*_3,3_	*HMM*_3,4_	**Recall (%)**
*G*_3,1_	31	2	1	1	88.57
*G*_3,2_	0	33	1	0	97.05
*G*_3,3_	2	2	16	1	76.19
*G*_3,4_	0	0	0	27	100
**Precision (%)**	93.93	89.18	88.88	93.1	
	${HMM}_{3,1}^{SS}$	${HMM}_{3,2}^{SS}$	${HMM}_{3,3}^{SS}$	${HMM}_{3,4}^{SS}$	**Recall (%)**
*G*_3,1_	31	2	1	1	88.57
*G*_3,2_	0	33	1	0	97.05
*G*_3,3_	1	1	17	2	80.95
*G*_3,4_	0	0	0	27	100
**Precision (%)**	96.87	91.66	89.47	90	
	3*DCNN*_3,1_	3*DCNN*_3,2_	3*DCNN*_3,3_	3*DCNN*_3,4_	**Recall (%)**
*G*_3,1_	35	0	0	0	100
*G*_3,2_	0	27	7	0	79.4
*G*_3,3_	2	2	17	0	80.9
*G*_3,4_	0	0	0	27	100
**Precision (%)**	94.5	93.1	70.8	100	

*GV*_4_ consists of five different gestures. The clusters used in the *k*-means approach in combination with an HMM with 12 hidden states were 25. The proposed methodology in this work performed better than the rest of the machine learning methods, with *f*-score results improved by +12% (Table 6).

**Table 6:** Confusion matrix using *HMM* and *HMM*^*SS*^ approach for *GV*_4_.

	*HMM*_4,1_	*HMM*_4,2_	*HMM*_4,3_	*HMM*_4,4_	*HMM*_4,5_	**Recall (%)**
*G*_4,1_	42	1	0	0	1	95.4
*G*_4,2_	3	86	0	0	1	95.5
*G*_4,3_	0	0	75	2	12	84.2
*G*_4,4_	1	0	0	43	0	97.7
*G*_4,5_	2	0	3	0	75	93.7
**Precision (%)**	87.5	98.8	96.15	95.5	86.2	
	${HMM}_{4,1}^{SS}$	${HMM}_{4,2}^{SS}$	${HMM}_{4,3}^{SS}$	${HMM}_{4,4}^{SS}$	${HMM}_{4,5}^{SS}$	**Recall (%)**
*G*_4,1_	42	1	0	0	1	95.5
*G*_4,2_	3	86	0	0	1	95.5
*G*_4,3_	0	0	87	0	2	89.8
*G*_4,4_	5	2	0	37	0	84
*G*_4,5_	1	0	5	0	74	92.5
**Precision (%)**	82.3	96.6	94.5	100	94.8	

In Table 7, the comparison of mean *f*-scores for each *GV* and each approach is presented. The score of *GV*_1_ and *GV*_2_ was improved by ~2%, while the most important contribution is observed for the *GV*_3_. The *HMM*^*SS*^ allows to improve significantly (+7.5%) the recognition results of this last dataset.

**Table 7:** Comparison of mean *f-scores* and final accuracies of each *GV* for *HMM* and *HMM*^*SS*^ approach.

**Mean f-score**	**Datasets**			
	*GV*_1_%	*GV*_2_%	*GV*_3_%	*GV*_4_%
*HMM*	94.34	83.1	90.64	92.1
*HMM^*SS*^*	96.21	85	91.57	92.29
*k −* means *+ HMM*	-	-	-	80
*3DCNN*	93.4	84	90	-
**Total accuracy**	**Datasets**			
	*GV*_1_%	*GV*_2_%	*GV*_3_%	*GV*_4_%
*HMM*	94.56	82.5	91.45	92.5
*HMM^*SS*^*	96.19	85	92.3	93.94
*k −* means *+ HMM*				82
*3DCNN*	93.5	87	89	

*For the datasets GV_1_, GV_2_ and GV_3_ also the 3DCNN results are presented, based on the model of Tran et al. (2015). For GV_4_ the results are compared to those presented in Coupeté et al. (2019) using a k-means and HMM approach, with 25 clusters and discrete HMMs with 12 hidden states*.

A similar conclusion can be extracted from the same table, where the total accuracy for the *GV*_3_ has reached 80.34% from 70.94%. The accuracy improvement of the two other datasets remain at the same level with the one of the mean *f*-score, around +2%.

In order to compare the results of the approach proposed in this paper with other classification techniques, a DL end-to-end 3D CNN has been used to classify the gestures of the three first vocabularies described in *Industrial Datasets and Gesture Vocabularies*. More precisely, a 3*DCNN* has been initially trained on spatiotemporal features from a medium-sized UCF-101 video dataset, and the pretrained weights have been used to fine-tune the model on small-sized datasets including images of operators performing customized gestures in industrial environments.

The architecture of the network is based on the one proposed in Tran et al. (2015) with four convolution and two pooling layers, one fully connected layer, and a softmax loss layer to predict action labels. It has been trained from scratch on the UCF-101^2^ video dataset, using batch size of 32 clips and the Adam optimizer (Kingma and Ba, 2014) for 100 epochs, with the Keras DL framework (Chollet, 2015). The entire network was frozen, and only four last layers were fine-tuned on customized datasets by backpropagation.

The comparison of recognition accuracy results between *HMMs*, *HMM*^*SS*^, and 3*DCNN* is shown in Tables 3, 7. As far as the *GV*_1_ is concerned, the use of a 3*DCNN* improves the recognition of only one gesture (*G*_1,1_) as shown in Table 3. However, in total, the *HMM*^*SS*^ outperforms the other two methods, reaching a total accuracy of 96.19% (Table 7). In the second dataset **(***GV*_2_), 3*DCNN* does not achieve a satisfying recognition result for the *G*_2,5_ (66%) in comparison with other methods that reach 100% (Table 4); and in total, *HMM*^*SS*^still performs the best as it is possible to observe in Table 7. In the *GV*_3_, the *HMM*^*SS*^performs again the best among the three methods, as shown also in Table 7, with a total accuracy almost +4% higher and an *f*-score of +1.5% higher than the DL method.

### Forecasting Ability for Motion Trajectories

For the evaluation of the ability of the four SS models that are used to explain the assumptions of the two-entity GOM, a simulation using Equation (2) for all three dimensions and for all used joints was performed (Table 8). It includes the computation of Theil's inequality coefficient (*U*) and its decomposition into the inequality of bias proportion *U*^B^, variance proportion *U*^V^, and covariance proportion *U*^C^. *U*^B^ examines the relationship between the means of the actual values and the forecasts, *U*^V^ considers the ability of the forecast to match the variation in the actual series, and *U*^*C*^ captures the residual unsystematic element of the forecast errors (Wheelwright et al., 1997). Thus, *U*^B^ + *U*^V^ + *U*^C^ = 1. The Theil inequality coefficient measures how close the simulated variables are to the real variables, and it gets values from 0 to 1. The closer to 0 the value of this factor is, the better the forecasting of the variable. Also, the forecasting ability of the model is better when *U*^B^ and *U*^V^ are close to 0 and *U*^C^ is close to 1. The computed coefficients as shown in Table 8 and result to a sufficient forecasting performance of the simulated model, and the error results reinforce this conclusion. Also, because the *U*^V^ values are really very close to 0, we could extract the potential conclusion that model is able to forecast efficiently even when the real motion data vary significantly (e.g., different operators).

**Table 8:** Theil inequality coefficient, root mean squared error, for one example of the X coordinate of the right wrist per dataset.

**Gestures**	**Theil Inequality *U***	**Bias proportion *U*^**B**^**	**Variance proportion *U*^**V**^**	**Covariance proportion *U*^**C**^**	**RMSE**
*G*_*1,1*_	0.018388	0.009178	0.081456	0.909366	0.028904
*G*_*2,1*_	0.0000373	0	0.017247	0.983653	0.007461
*G*_*3,1*_	0.0000161	0	0.008713	1.041715	0.003277
*G*_*4,1*_	0.010059	0	0.039551	0.960449	0.018053

**Figure 6:**
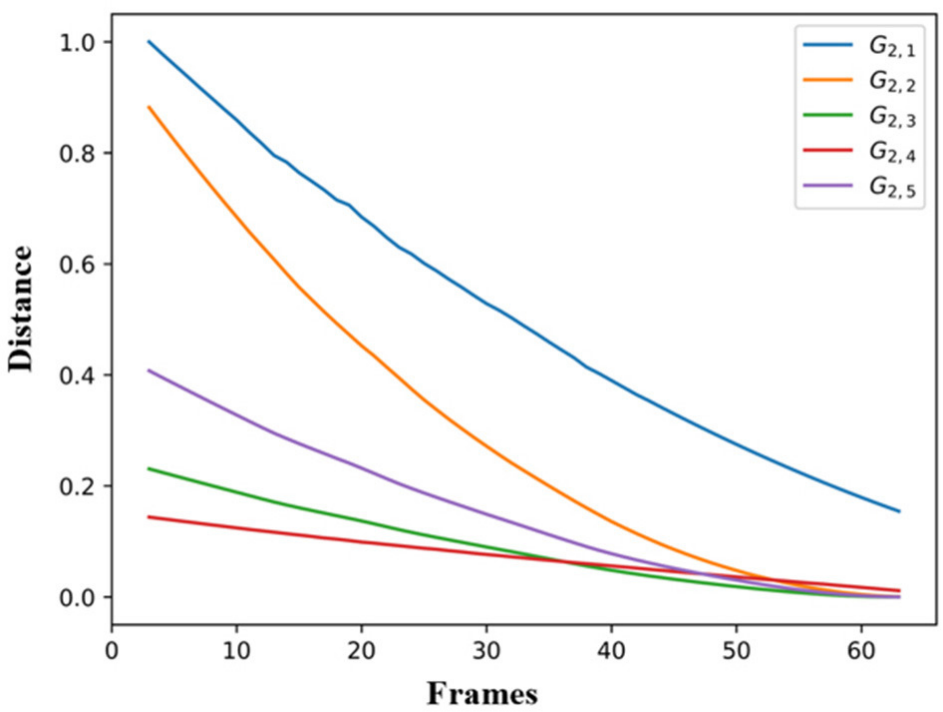
Forecasting performance of all the SS models of *GV*_2_ on the variable *RWRIST*_*X*_ based on *G*_2,4_.

Finally, Figure 6 presents an example of trajectory forecasting for *GV*_2_. More specifically, the forecasting performance of all the SS models of GV_2_ on the variable *RWRIST*_*X*_ is presented, when an unknown observation with data from the *G*_2,1_ is provided to them. The similarity or distance metric from the DTW is plotted on Figure 6 taking as input for every time t: 1) the simulated values of the *RWRIST*_*X*_ on *G*_2,4_ when providing it with real observations until *t* = 1 (starting from *t* = 3), and 2) the real observations between *t* and the end of the sequence. The distance becomes minimum (high similarity) from the very first time-stamp for the SS model of *G*_2,4_.

### Sensitivity Analysis

As mentioned, the GOM depicts all the possible relationships that take place during the process of the performance of a gesture. Following the GOM, the next steps are the estimation of the model, its dynamic simulation, and its sensitivity analysis. All those steps lead to checking the model's structure, forecasting ability, and its reaction to shocks of its variables, respectively.

**Figure 7:**
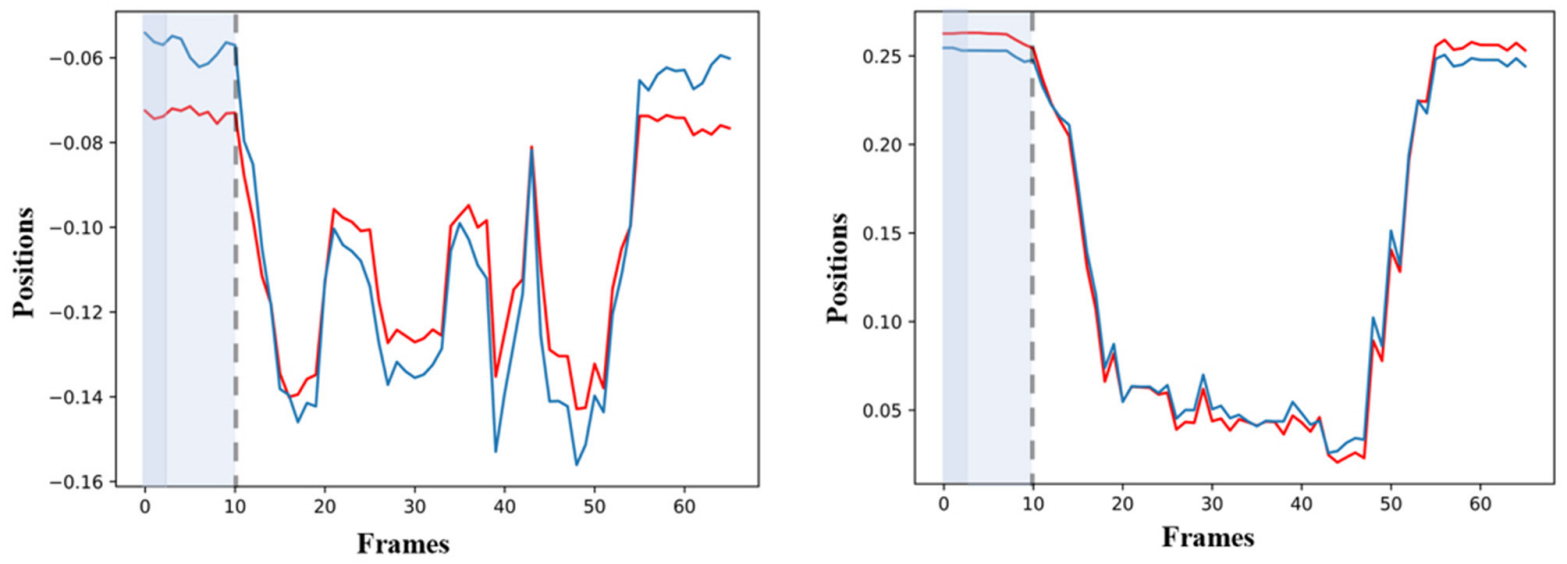
Left: Diagram of the simulated forecasted values of *RWRIST*_*X*_ before the disturbance (red) and simulated forecasted values of *RWRIST*_*X*_ (blue), after the shock on the values of *RWRIST*_*Y*_ by 80% for two frames. Right: Diagram of the simulated forecasted values of *RWRIST*_*Y*_ before the disturbance (red) and simulated forecasted values of *RWRIST*_*Y*_ after the shock on the values of *RWRIST*_*X*_ by 80% for two frames.

The sensitivity analysis of the simulated GOM follows two steps. During the first step, all the simulated values of the model are being estimated, after an artificial shock is provoked for the first two frames. In the second step, all the simulated values that came up after the disturbance are being compared with the simulated values before it (baseline). For example, in Figure 7, the simulated values of *RWRIST*_*X*_ are depicted before (red color) and after (blue color) the disturbance on the values of *RWRIST*_*Y*_ by 80%. The disturbance on the simulated variables of *RWRIST*_*X*_ is observed for 10 frames in total, eight more frames than the duration of the initial shock. A similar behavior is also observed for *RWRIST*_*Y*_. The quick adaptation of the models after the application of the artificial shock is observed, which also confirms the low sensitivity of the models to external disturbances.

## Discussion

The proposed method for human movement representation on multivariate time series has been used for recognition of professional gestures and forecasting of their trajectories. A comparison has been done between the recognition results of our hybrid approach and the standard continuous HMMs. In general, with both approaches, the best recognition accuracy is achieved for the *GV*_1_. This can be explained by the beneficial *inter*class and *intra*class variations of this vocabulary. The gestures are sufficiently discrete, whereas the different repetitions performed by one operator are sufficiently similar. Nevertheless, we observe an improvement on the recognition accuracy for micro-gestures, when the confidence control of the *HMM*^*SS*^ is applied for micro-movements, for example, assembling small pieces, whereas the performance of *HMM*s is satisfactory for macro-movements.

The second-best results are given for the *GV*_2_. Even though these gestures are simpler and do not require any particular dexterity, less good results in recognition accuracy in comparison with the *GV*_1_ are expected mostly because of the high intraclass variation due to multiple users. Although they followed a protocol, each person had significant variations in the way he/she performed the commands. For both datasets, a slight improvement of results has been achieved.

As explained in *Industrial Datasets and Gesture Vocabularies*, the biggest difference of the *GV*_3_ in comparison with the other two gesture vocabularies is the low *inter*class variation because the gestures are similar between them. In three out of four gestures, common gestural patterns are presented: the glass master if controlling the pipe with the left hand is manipulating the glass with the right while sitting, and so forth. These common gestural patterns generate the low *intra*class variation. This low variation can be due to the high level of expert's dexterity, the use of a predefined physical setup (metallic construction) that places his/her body and gestures in a spatial framework (situated gestures) and the use of professional tools that also reduces potential freedom in gesture performances. The low *intra*class variation is also underlined by the comparison of the *RMSE* values for different repetitions of the same gesture performed by the same person. The *HMMs* are thus expected to provide less good results among the four datasets, for the *GV*_3_, because this method may struggle in managing low interclass variation. An important similarity between classes is expected to augment the uncertainty in the maximum likelihood probabilities given by the *HMMs*. This hypothesis can be confirmed through the current recognition results on the basis of *HMMs*. However, it can be clearly noticed that *HMM*^*SS*^ had the most beneficial impact on the recognition accuracy of the *GV*_3_. A conclusion can be thus formulated that the proposed methodology permits the improvement of the gesture recognition results to a significant level.

The recognition results of all the three gestural vocabularies using machine learning methods were also compared with those when using 3*DCNNs* as a DL method for gesture recognition. In all of the three experiments, the *HMM*^*SS*^ method outperformed, and especially in *GV*_1_, achieved +3% higher *f*-score and accuracy compared with the 3*DCNNs*. In the gestural vocabularies *GV*_1_ and *GV*_3_, the *HMM* method, even if it was not combined with the SS method, achieved slightly higher *f*-score results than the 3*DCNNs*.

As far as the *GV*_4_ is concerned, our current approach of continuous *HMMs* and SS outperforms our previous one that used *k*-means and discrete *HMMs* (Table 7). More precisely, an improvement of at least +12% is observed on the mean *f*-score, together with an improvement of at least +10% at the total accuracy.

As far as the ability of the models to effectively simulate the professional gestures is concerned, the graphical depiction of the simulated values of the models together with the real motion data can lead to encouraging conclusions. Initially, the simulated values follow very well the real ones for the whole gesture. In particular, the results on *GV*_1_ are quite promising because the pose estimation had some fails because of the top-mounted camera. Nevertheless, the changes or discontinuities on the motion data did not affect the simulation ability of the models. With the regard to the forecasting ability of the models, it is obvious that if the model follows the trajectory from the very beginning, then its forecasting ability is maximized, which is the case in Figure 6. Moreover, the evaluation of the forecasting ability of the models using the coefficient of Theil is also encouraging, thus opening a possibility for an efficient forecasting of motion trajectories. In parallel, the sensitivity analysis applied to equations variables proves forecasting's ability of the model to react rapidly to shocks and to provide a solid prediction of motion trajectories.

## Conclusion and Future Work

In this paper, a gesture operational model is proposed to describe how the body parts cooperate to perform a professional gesture. Several assumptions are formulated that determine the dynamic relationship between the body entities within the execution of the human movement. The model is based on the SS statistical representation, and a simultaneous equation system for all the body entities is generated, which is composed of a set of first-order differential equations. The coefficients of the equation system are estimated using the MLE, and its simulation generates a tolerance of the spatial variance of the movement over time. The scientific evidence of the *GOM* is evaluated through its ability to improve the recognition accuracy of gestural time series that are modeled using continuous HMMs. Four datasets have been created for this experiment, corresponding to professional gestures from industrial real-life scenarios. The proposed approach overperformed the recognition accuracy of the *HMMs* by approximately +2% for two datasets, whereas a more significant improvement of +10% has been achieved for the third dataset with strongly situated professional gestures. Furthermore, the approach has been compared with an end-to-end 3DCNN approach, and the mean *f*-score of the proposed method is significantly higher than the DL, varying approximately from +1.57 to +2.9% better performance, depending on the dataset. A second comparison is done by using a previously recorded industrial dataset from a human–robot collaboration. The proposed approach gives ~ +13% for the mean *f*-score and +12% for total accuracy, compared with our previous hybrid *k*-means and discrete HMM approach.

Moreover, the system is simulated through the solution of its equations. Its forecasting ability has been evaluated by comparing the similarity between the real and simulated motion data, using at least two real observations to initialize the system, as well as by measuring the Theil inequality coefficient and its decompositions. This paper opened a potential for investigating simultaneous real-time probabilistic gesture and action recognition, as well as forecasting of human motion trajectories for accident prevention and very early detection of the human intention. Therefore, our future work will be focused on extending the proposed methodology for real-time recognition and enhancing the GOM to include kinetic parameters as well. Finally, there will be a continuous enrichment of the datasets by adding new users and more iterations.

## Data Availability Statement

The datasets generated for this study will not be made publicly available. The datasets generated for this study are anonymous and by the time of the article submission the authors do not have authorization from the industries to publish them. Negotiation is being done with the companies and organizations involved to make the data publicly available.

## Ethics Statement

Written informed consent was obtained from the individual(s), and minor(s)' legal guardian/next of kin, for the publication of any potentially identifiable images or data included in this article.

## Author Contributions

SM: Conceptualisation of the methodology and definition of the scientific and industrial needs to address with respect to the human motion analysis, machine learning, and pattern recognition. GS: Contribution to the recording of the dataset, implementation of the experiments, and configuration of the statistical parameters of State-Space. DM: Leading the recording of the datasets, pose estimation and festure selection, developing and integrating the algorithms, and MLE and kalman filtering fine tuning the forecasting ability of the models. AG: Definition of the protocol for the recordings, human factor analysis of the motion data and definition of the vocabularies, and interpretation of the recognition results.

## Conflict of Interest

The authors declare that the research was conducted in the absence of any commercial or financial relationships that could be construed as a potential conflict of interest.

## Footnotes

^1^https://github.com/CMU-Perceptual-Computing-Lab/openpose/blob/master/doc/output.mdwhwhoch

^2^https://www.crcv.ucf.edu/data/UCF101.php
