# Erratum: Human Movement Representation on Multivariate Time Series for Recognition of Professional Gestures and Forecasting Their Trajectories

**DOI:** 10.3389/frobt.2020.639181

**Published:** 2020-12-16

**Authors:** Frontiers Production Office

**Affiliations:** Frontiers Media SA, Lausanne, Switzerland

**Keywords:** state-space representation, differential equations, movement modeling, hidden Markov models, gesture recognition, forecasting, motion trajectory

Due to a production error, several words were incorrectly capitalized throughout the text. These have now been correctly capitalized.

In **Equations 17–20**, there were several instances where the authors mistakenly switched the positive and negative signs.

In the **Statistical Significance and Simulation of the Models** section, subsection **Recognition Accuracy and Comparison and End-to-End Deep Learning Architectures**, paragraph 12, the authors accidently missed the addition of a footnote. The following footnote has been added for UCF-101: https://www.crcv.ucf.edu/data/UCF101.php.

In **Table 7**, “Kingma and Ba (2014)” should be replaced with “Tran et al. (2015).” Following that, “Tran et al. (2015)” should be replaced with “Competé et al. (2019).”

In the legend for **Figure 6**, a sentence has been added to clarify that this is based on “G_2,4_” where 2 & 4 are pointers.

In the **Statistical Significance and Simulation of the Models** section, subsection **Forecasting Ability for Motion Trajectories**, paragraph 2, The final two mentions of “G_2,1_” have been corrected to “G_2,4_.”

The publisher and authors apologize for these errors. The original version of this article has been updated.

